# The mRNA repressor TRIM71 cooperates with Nonsense-Mediated Decay factors to destabilize the mRNA of CDKN1A/p21

**DOI:** 10.1093/nar/gkz1057

**Published:** 2019-11-16

**Authors:** Lucia A Torres-Fernández, Bettina Jux, Maximilian Bille, Yasmine Port, Karin Schneider, Matthias Geyer, Günter Mayer, Waldemar Kolanus

**Affiliations:** 1 Molecular Immunology and Cell Biology, Life & Medical Sciences Institute (LIMES), University of Bonn, 53115 Bonn, Germany; 2 Institute of Structural Biology, University Clinics Bonn, University of Bonn, 53127 Bonn, Germany; 3 Center of Aptamer Research & Development; Chemical Biology & Chemical Genetics, Life & Medical Sciences Institute (LIMES). University of Bonn, 53121 Bonn, Germany

## Abstract

Nonsense-mediated decay (NMD) plays a fundamental role in the degradation of premature termination codon (PTC)-containing transcripts, but also regulates the expression of functional transcripts lacking PTCs, although such ‘non-canonical’ functions remain ill-defined and require the identification of factors targeting specific mRNAs to the NMD machinery. Our work identifies the stem cell-specific mRNA repressor protein TRIM71 as one of these factors. TRIM71 plays an essential role in embryonic development and is linked to carcinogenesis. For instance, TRIM71 has been correlated with advanced stages and poor prognosis in hepatocellular carcinoma. Our data shows that TRIM71 represses the mRNA of the cell cycle inhibitor and tumor suppressor CDKN1A/p21 and promotes the proliferation of HepG2 tumor cells. CDKN1A specific recognition involves the direct interaction of TRIM71 NHL domain with a structural RNA stem-loop motif within the CDKN1A 3′UTR. Importantly, CDKN1A repression occurs independently of miRNA-mediated silencing. Instead, the NMD factors SMG1, UPF1 and SMG7 assist TRIM71-mediated degradation of CDKN1A mRNA, among other targets. Our data sheds light on TRIM71-mediated target recognition and repression mechanisms and uncovers a role for this stem cell-specific factor and oncogene in non-canonical NMD, revealing the existence of a novel mRNA surveillance mechanism which we have termed the TRIM71/NMD axis.

## INTRODUCTION

Nonsense-mediated decay (NMD) is an important RNA surveillance pathway well known to control the degradation of transcripts harboring premature termination codons (PTC) (1,2). Beyond its canonical role as a quality control pathway, NMD has emerged in recent years as a pathway which can also regulate the expression of functional transcripts (3,4). Therefore, NMD not only plays an essential role in preventing the production of truncated proteins that could have deleterious effects on the organism, but it also impacts a wide range of physiological processes, such as differentiation and development, response to stress, immune response, proliferation and cancer (5,6).

Induction of NMD for a particular transcript is linked to the interpretation of a premature translation termination (7), and therefore, discerning between a PTC and the normal stop codon is crucial for eliciting canonical NMD. For most transcripts, the normal stop codon lies within the last exon. After pre-mRNA splicing, a group of proteins known as the exon junction complex (EJC) remain bound to the mRNA 20–25nt upstream of the exon-exon junctions, and EJCs are then displaced by the ribosome during the first round of translation (8,9). PTCs are thereby marked by EJCs typically located more than 50–55nt downstream of the PTC (10). When the ribosome stalls at a PTC, the major NMD effector UPF1 together with its activating kinase SMG1 are recruited through their binding to the release factors eRF1 and eRF3 to form the surveillance complex (SURF). Subsequently, the SURF complex interacts with other NMD effectors present in the EJC – UPF2 and UPF3b – to form the decay-inducing complex (DECID), resulting in SMG1 activation and UPF1 phosphorylation. Phosphorylated UPF1 recruits both the endonuclease SMG6, which cleaves the RNA in the vicinity of the PTC, and the dimer SMG5–SMG7, which triggers CCR4–NOT-mediated deadenylation and DCP2-mediated decapping. The downstream RNA products are then subjected to 3′–5′ and 5′–3′ exonucleolytic decay by the exosome complex and XRN1, respectively (5).

The EJC-dependent model explains how NMD operates in PTC-containing transcripts, which include not only aberrant transcripts resulting from nonsense mutations, but also transcripts with alternative reading frames, transcripts with introns in their 3′UTR yielding a PTC-like situation, transcripts resulting from alternative splicing or programmed ribosomal frameshifts, and transcripts encoding for selenoproteins, in which the stop codon UGA can be redefined to encode for selenocysteine in a high selenium environment (6). However, NMD suppression upregulates many transcripts lacking all of these features (11), and EJC-independent NMD mechanisms have been previously reported (12). A long 3′UTR is a common feature of PTC-lacking NMD targets (13,14), although neither UTR length nor any of the aforementioned RNA features *per se* guarantees a reliable prediction of NMD targets (11,15). Therefore, the signals and factors recruiting the NMD machinery to PTC-lacking mRNAs remain to be identified for specific targets and cellular contexts. The present work identifies the stem cell-specific mRNA-binding protein TRIM71 as a factor cooperating with the NMD machinery to repress the expression of its specific target CDKN1A, as well as other mRNAs.

TRIM71/LIN41 was first identified as an heterochronic gene controlling developmental timing in *Caenorhabditis elegans (*16). Its expression is conserved among all metazoans and is restricted to undifferentiated stem and progenitor cells during early embryonic development (17), correlating temporally with proliferative and pre-differentiation processes and spatially with the pluripotency factor OCT4 (18). Despite its short expression window, TRIM71 is essential for embryonic development (17). Embryonic lethality in mice is accompanied by neural tube closure defects (19). Within the mouse embryo, TRIM71 locates to brain, muscle and gonads (16). Conditional loss of TRIM71 in those organs has been shown to lead to exencephaly in mice (19), loss of muscle attachment in fruitflies (20) and sterility in nematodes (16). Thus, TRIM71 is not only essential for embryonic development but presumably required for the proper function of these organs in the adult organism.

TRIM71 belongs to the TRIM-NHL protein family, whose versatile domain structure includes a RING domain followed by two B-Boxes (BB), a Coiled-Coil region (CC), a Filamin domain (FLN) and a NHL domain consisting of six β-propeller repeats (21). Its RING domain enables TRIM71 to act as an E3 ligase whereas the NHL domain is involved in its role as an mRNA repressor (17). Some examples of proteins ubiquitylated by TRIM71 are the adaptor protein SHCBP1, which is involved in FGF signalling (22,23), or the pro-apoptotic tumor suppressor p53 (24). MRNAs repressed by TRIM71 include the cell cycle inhibitor and tumor suppressor CDKN1A/p21 (25) and the differentiation-promoter transcription factor EGR1/Lin-29 (26). The array of protein and mRNA targets regulated by TRIM71 collectively justify its reported role in promoting proliferation and preventing differentiation in developmental and oncogenic processes (17). Although the causes of embryonic lethality remain unknown, most of the loss-of-function mutations in TRIM71 clustered in the NHL domain in *C. elegans* (16). Furthermore, a TRIM71 mutant merely lacking the last NHL repeat phenocopied a total loss of TRIM71 in a mouse model (19), underscoring the critical importance of its function as an mRNA repressor. Regarding its role in mRNA repression, it has been proposed that TRIM71 cooperates with miRNAs based on its localization in P-bodies, its interaction with proteins involved in the miRNA pathway, such as AGO1, AGO2, AGO4 and DICER, and the fact that TRIM71 shares targets with miRNAs (18,25,27). Furthermore, TRIM71-deficient murine embryonic stem (mES) cells show an altered miRNA landscape, with ES-specific miRNAs being reduced at the expense of increased differentiation-promoting miRNAs, namely brain- and gonad-specific miRNAs (28). Altogether, this is highly indicative of a role for TRIM71 in the regulation of miRNA biogenesis, but whether TRIM71 requires miRNA assistance for specific mRNA repression remains elusive.

In order to gain molecular insight into the role of TRIM71 as an mRNA repressor, the present work focuses on the regulation of the known TRIM71 mRNA target CDKN1A (25), and its involvement in hepatocellular carcinoma cell proliferation. Our results show that TRIM71 directly interacts with a specific structural RNA motif present in the CDKN1A 3′UTR and induces target degradation in cooperation with NMD factors. Our work uncovers the TRIM71/NMD axis, a novel RNA surveillance mechanism enabling the participation of NMD in the degradation of functional transcripts.

## MATERIALS AND METHODS

### Cell culture

HepG2 cells were cultured in RPMI 1640 media supplemented with 10% FBS and 1% Penicillin–Streptomycin antibiotic solution. HEK293T cells were cultured in DMEM media supplemented with 10% FBS and 1% Penicillin-Streptomycin antibiotic solution. HEK293 cells were cultured in DMEM media supplemented with 10% FBS and 600 μg/ml of G418-BC antibiotic solution. mES cells were cultured in DMEM knockout media supplemented with 15% FCS, 1% Penicillin-Streptomycin, 1% non-essential amino acids, 1% GlutaMAX, 0.2% LIF and 0.1% β-mercaptoethanol. All cells were grown by incubation at 37°C in a controlled atmosphere with 5% CO_2_ and 95% relative humidity and split when 60–70% confluence was reached (see Cell Reagents table in Supplementary Methods). The generation of stable GFP-Ctrl/GFP-TRIM71-overexpressing HEK293 cells and AGO2 knockout HEK293T cells is described in Supplementary Methods.

### Plasmids and transfections

Directional cloning of ORF sequences in prk5-FLAG, pN1-Ig and pN1-GFP vectors for protein overexpression was performed with primers containing MluI/NotI restriction sites. Directional cloning of 3′UTR sequences in psiCHECK2 vectors for luciferase reporter assays was performed with primers containing XhoI/NotI restriction sites (see Cloning Primers table in Supplementary Methods). HEK293T cells were transfected using a calcium phosphate solution containing 25 μg DNA/ml. mES cells were transfected using PANfect reagent at a ratio of 1μg:2μl DNA:PANfect. HepG2 cells were transfected using Lipofectamine2000 reagent at a ratio of 1μg:1μl DNA:Lipofectamine. RNA transfection was performed with Lipofectamine RNAiMAX reagent for all cell lines, using a ratio of 10pmol:2μL RNA/Lipofectamine (see Cell Reagents table in Supplementary Methods). Cells transfected with DNA for protein overexpression were harvested 48 hours post-transfection (hpt), while cells transfected with siRNA for protein knockdown were harvested 72 hpt (see siRNAs and miRNAs tables in Supplementary Methods).

### Proliferation assays

Cells were stained with the proliferation dye eFluor670 (see Cell Reagents table in Supplementary Methods), diluted to 5 μM in PBS (1 ml of PBS per 10^6^ cells), and seeded in triplicates. The initial fluorescence intensity (day 0) and fluorescence decrease upon cell division over time were monitored by FACS every 24 h for 4 days after staining (see Software table in Supplementary Methods). HepG2 cells were transfected with siRNAs 24 h before staining. The number of cell divisions was calculated assuming a decrease of the median fluorescence intensity (MFI) by half upon each cell division with the following formula: division number = log_2_ [(MFI_day0_ – MFI_unstained_)/(MFI_dayX_ – MFI_unstained_)]. The average cell cycle duration was estimated at the end of the experiment from the number of cell divisions.

### Protein extraction and Western blotting

Cell pellets were lysed in RIPA Buffer (20 mM Tris–HCl pH 7.5, 150 mM NaCl, 1 mM Na_2_EDTA, 1 mM EGTA, 1% NP-40, 1 mM Na_3_VO_4_, 1% sodium deoxycholate, 2.5 mM sodium pyrophosphate, 1 mM β-glycerophosphate) supplemented with protease inhibitors (see Enzymes & Inhibitors table in Supplementary Methods), and protein lysates were pre-cleared by centrifugation and quantified using the Pierce BCA assay kit according to the manufacturer's instructions (see Commercial Kits table in Supplementary Methods). Protein lysates were then denatured by incubation with SDS Buffer (12% glycerol, 60 mM Na_2_EDTA pH 8, 0.6% SDS, 0.003% bromophenol blue) for 10 min at 95°C and separated by PAGE-SDS in Laemmli Buffer (25 mM Tris, 192 mM glycine, 0.1% SDS). Proteins were then wet-transferred to a nitrocellulose membrane in transfer buffer (25 mM Tris–HCl pH 7.6, 192 mM glycine, 20% methanol, 0.03% SDS) and membranes were then blocked with 3% BSA in 1× TBST (50 mM Tris–HCl pH 7.6, 150 mM NaCl, 0.05% Tween-20) prior to overnight incubation at 4°C with the required primary antibodies (see Antibodies table in Supplementary Methods). After washing the membrane three times with 1× TBST, they were incubated with a suitable HRP-coupled secondary antibody for 1 h at RT followed by three washing steps with 1× TBST. Membranes were developed with Pierce ECL substrate kit according to the manufacturer's instructions (see Commercial Kits table in Supplementary Methods).

### Protein immunoprecipitation (IP)

Proteins were extracted in IP-buffer (10 mM HEPES pH 7.5, 2 mM MgCl_2_, 10 mM KCl, 0.5% NP-40, 0.5 mM EDTA, 150 mM NaCl, 1 mM DTT) supplemented with protease inhibitors. Protein lysates were pre-cleared by centrifugation, quantified using the Pierce BCA assay kit, and at least 500 μg of protein (1 μg/μl) were incubated with 10 μl of magnetic beads for 6 h at 4°C on a rotating wheel. A portion of each whole cell lysate was retained as an input control. ANTI-FLAG M2 magnetic beads were used for the immunoprecipitation of Flag-tagged proteins, while Protein G Dynabeads were used for the immunoprecipitation of IgG-tagged proteins or pre-coupled to suitable antibodies (diluted to 50–100 ng/μl in IP buffer) for the IP of endogenous proteins (see Antibodies table in Supplementary Methods). Antibody coupling was performed for 2 h at 4°C on a rotating wheel prior to their incubation with the lysates. After IP, the beads were washed five times with 1 ml IP-buffer and bound proteins were eluted from the beads by boiling in SDS buffer, followed by PAGE-SDS separation and western blotting as described above. For RNase treatment, washed IP fractions were incubated with 200 μg/ml RNase A (see Enzymes & Inhibitors table in Supplementary Methods) for 30 min at 37°C and washed three more times with IP-buffer before protein elution.

### UV-Crosslinking RNA-immunoprecipitation (RNA-IP)

For RNA immunoprecipitation, cells were carefully washed with cold PBS so that they remained adherent, and open dishes were irradiated once with UV light at 300 mJ/cm^2^ to crosslink nucleic acids to their protein binding partners. Cells were then harvested and lysed in 500 μl RNA-IP buffer ((TKM buffer (20 mM Tris pH 7.4, 100 mM KCl, 5 mM MgCl_2_), supplemented with NP-40 (0.2%), RNase inhibitor (120 U/ml) and protease inhibitors (see Enzymes & Inhibitors table in Supplementary Methods)). Cell lysates were pre-cleared by centrifugation, quantified using the Pierce BCA assay kit, and 2 mg of protein (2 μg/μl) were incubated with 30 μl of magnetic beads overnight at 4°C on a rotating wheel. A portion of each whole cell lysate was retained as an input protein control (total protein) and an input RNA control (total RNA). After RNA-IP, beads were washed five times with RNA-IP buffer and 20% of the IP lysate was used for western blot analysis (IP protein), while 80% of the IP lysate was digested with 500 μg/ml proteinase K (see Enzymes & Inhibitors table in Supplementary Methods) for 30 min at 37°C and used for RNA extraction followed by qPCR (IP RNA). For a given target, quantified IP RNA values were normalized to total RNA values to calculate the IP enrichment, and the IP enrichment of each mRNA target was normalized to the unspecific IP enrichment of the housekeeping 18S rRNA.

### RNA extraction and real-time semiquantitative PCR (qPCR)

RNA was extracted from cell pellets using the Trizol-containing reagent peqGold TriFAST according to the manufacturer's instructions. RNA pellets were resuspended in RNase-free water, and DNA digestion was performed prior to RNA quantification. 0.5–1 μg of RNA were reverse transcribed to cDNA using the High Capacity cDNA Reverse Transcription Kit according to the manufacturer's instructions. The cDNA was then diluted 1:5, and a relative quantification of specific genes was performed in a Bio-Rad qCycler using either TaqMan probes in TaqMan master mix or specific primer pairs in SYBR-Green master mix (see Commercial Kits and qPCR primers tables in Supplementary Methods).

### Confocal microscopy imaging

For P-body colocalization studies, HEK293T cells were cotransfected with pN1-RFP-DCP1A and the specified pN1-GFP construct at a 1:1 ratio and seeded on glass coverslips coated with 100 μg/μl poly-l-lysine 24 hpt. Cells were prepared for imaging 48 hpt by fixation in 4% PFA for 20 min at RT, followed by three PBS washing steps and then DAPI nuclear staining (diluted 1:1000 in PBS) for 60 min in a light-protected, humid chamber. The coverslips were then washed another three times with PBS and mounted on microscopy slides using FluoroShield mounting media containing 50 mg/ml Dabco. For DNA damage studies, 72 h after siRNA transfection, cells growing on coverslips were fixed, permeabilized with 0.2% Triton X-100 for 15 min at RT, blocked with 1% BSA/PBS for 1 h at RT and stained overnight with anti-phospho-H2AX antibody diluted 1:400 in blocking solution. After three PBS wash steps, simultaneous DAPI and Alexa647-coupled secondary antibody (1:400) staining was performed for 1 h at RT in the dark. Following three PBS washing steps, coverslips were mounted for imaging as specified above. For imaging, Olympus LSM FV-1000 and Zeiss LSM 880 microscopes were used in confocal mode with a 60× oil immersion objective.

### Luciferase assays

In overexpression studies, cells were cotransfected with the suitable psiCHECK2 plasmid and the specified pN1-Flag construct in a 1:4 ratio. In knockdown studies, psiCHECK2 plasmids transfections were performed 24 h after siRNA transfection. The psiCHECK2 plasmids contain a specific 3′UTR sequence downstream of the Renilla Luciferase ORF, while the Firefly Luciferase was used as a normalization control. To control for the possible 3′UTR-independent effects of any individual condition/construct on the psiCHECK2 vector backbone, a psiCHECK2 vector with no insert downstream of the Renilla sequence (Renilla-empty) was used. Transfected cells were harvested 48 h after psiCHECK2 plasmid transfection, and Renilla and Firefly signals were measured separately with the use of a luminometer and the Dual Luciferase Reporter Assay Kit according to the manufacturer's instructions (see Software and Commercial Kits tables in Supplementary Methods). For each condition, the Renilla-3′UTR/Firefly ratio was calculated and normalized to the Renilla-empty/Firefly ratio. The resultant values were specified as Normalized Relative Light Units (Norm. RLU).

### Fluorescence polarization assays

The capability of TRIM71 to directly bind RNA was evaluated by measuring changes in the polarization of 5′-Cy3-conjugated 13-mer ssRNAs (100 nM) upon binding with increasing concentrations of human recombinant Flag-TRIM71-NHL protein (see ssRNAs table in Supplementary Methods). Samples were brought to the equilibrium in 1× TBST buffer for 30 min at RT, and polarization was measured using a fluorescent plate reader equipped with polarizers. Binding did not alter the fluorescent intensity of the ssRNAs. The resultant binding-saturation curves were adjusted by non-linear regression using GraphPad Prism7 to estimate the equilibrium dissociation constant (*K*_d_). Recombinant protein production and purification, as well as protein structure computational modeling are described in Supplementary Methods.

### mRNA stability measurements

HepG2 cells were treated with 5 μg/ml of Actinomycin D for transcriptional inhibition, and samples for RNA extraction followed by qPCR measurement were taken 1, 2, 4 and 6 h after treatment initiation. Samples treated with DMSO were used as 0 h control. For a given target, the quantified expression of treated samples was normalized to the expression of the control sample and expressed as a percentage to depict mRNA decay rate over time. The resulting decay curves were adjusted by non-linear regression using GraphPad Prism7 to estimate the mRNA half-life (*t*_1/2_).

### Statistical analysis

Statistical significance was calculated with a two-tailed unpaired *t* test (ns = non-significant; *P-value < 0.05; **P-value < 0.01; ***P-value < 0.005) typically comparing a given condition sample to the control sample. In any other case, the compared samples are linked in graphs by a line. Regression curves analysis and area under curve (AUC) measurements were done with GraphPad Prism7.

## RESULTS

### TRIM71 regulates CDKN1A mRNA and promotes proliferation in HepG2 and HEK293 cells

**Figure 1.**
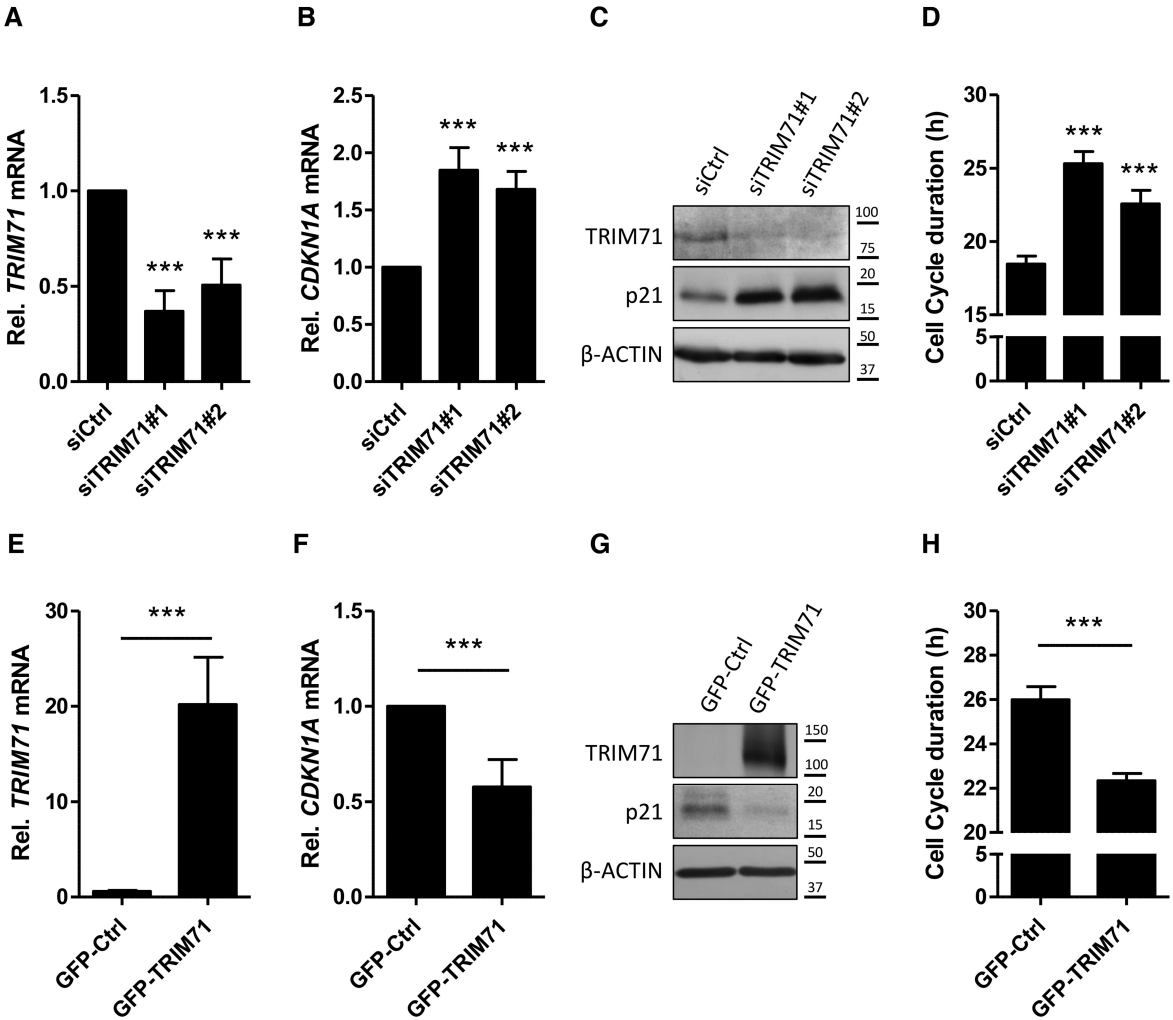
TRIM71 regulates CDKN1A mRNA and promotes proliferation in HepG2 and HEK293 cells. (**A**) TRIM71 and (**B**) CDKN1A mRNA levels measured by qPCR in control (siCtrl) and TRIM71 knockdown (siTRIM71#1 and #2) HepG2 cells 72 hpt (*n* = 6). (**C**) Representative immunoblot showing TRIM71 and CDKN1A/p21 protein levels in HepG2 cells upon TRIM71 knockdown, corresponding to mRNA levels from A and B. (**D**) Average cell cycle duration in hours (h), calculated from the number of cell divisions reached at day 4 (see also Supplementary Figure S3). (**E**) TRIM71 and (**F**) CDKN1A mRNA levels measured by qPCR in control (GFP) and TRIM71-overexpressing (GFP-TRIM71) stable HEK293 cells (*n* = 4). (**G**) Representative immunoblot showing TRIM71 and CDKN1A/p21 protein levels in HEK293 cells upon stable TRIM71 overexpression, corresponding to mRNA levels from E and F. (**H**) Average cell cycle duration in hours (h), calculated from the number of cell divisions reached at day 4 (see also Supplementary Figure S4). For qPCRs, HPRT1 housekeeping gene was used for normalization. All graphs represent Mean±SD.

TRIM71 expression is upregulated in several cancer types (Supplementary Figure S1) and has been correlated with advanced tumor stages and poor prognosis (29,30). TRIM71 was shown to promote proliferation in several hepatocellular carcinoma (HCC) cell lines as well as liver cancer progression in mouse xenograft models (29). The authors of that work proposed that TRIM71 controls tumorigenesis though its E3 ligase role via AGO1/2 proteins destabilization, a mechanism that was also postulated in an earlier work (18). However, in line with later studies (22,25,27,28), we did not observe TRIM71-mediated changes in AGO2 stability in the cell lines tested, including the hepatocellular carcinoma line HepG2 (Supplementary Figure S2A). In a previous study, TRIM71 was shown to repress the mRNA of the cell cycle inhibitor and tumor suppressor CDKN1A/p21 in mES and embryonic carcinoma (EC) cells (25). Interestingly, the strong TRIM71 expression observed in patients with advanced-stage HCC negatively correlates with CDKN1A mRNA expression, while it positively correlates with AGO2 expression (Supplementary Figure S2B). In order to evaluate whether TRIM71 may promote proliferation in HCC via CDKN1A downregulation, we conducted proliferation assays in HepG2 cells upon TRIM71 knockdown. Here, we observed that decreasing TRIM71 levels with two different siRNAs (Figure 1A) resulted in CDKN1A mRNA upregulation (Figure 1B), p21 protein increase (Figure 1C) and a significant prolongation of HepG2 cell cycle causing a 22% (siTRIM71#2) and 37% (siTRIM71 #1) decrease in proliferation (Figure 1D and Supplementary Figure S3). Conversely, stable overexpression of GFP-TRIM71 in HEK293 cells (Figure 1E), which express low endogenous TRIM71 levels, resulted in decreased CDKN1A mRNA levels (Figure 1F), p21 protein downregulation (Figure 1G) and significantly shorter cell cycles (14% enhancement of proliferation) as compared to GFP control HEK293 cells (Figure 1H and Supplementary Figure S4). Our results show that TRIM71 regulates CDKN1A mRNA levels and promotes proliferation in HepG2 and HEK293 cells.

### TRIM71 directly and specifically interacts with the 3′UTR of CDKN1A mRNA and represses its expression

**Figure 2.**
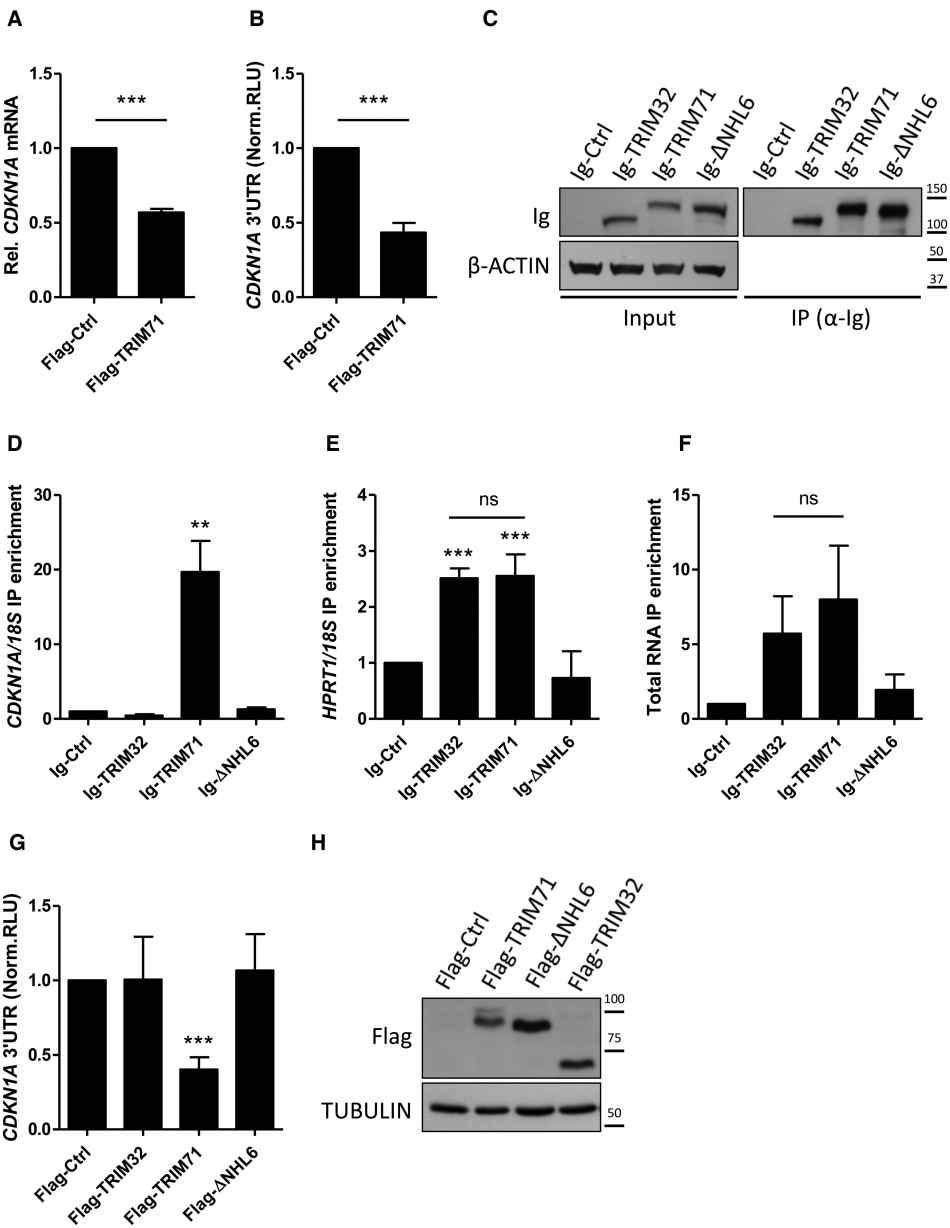
TRIM71 physically and specifically interacts with CDKN1A mRNA and represses its expression through the recognition of its 3′UTR. (**A**) CDKN1A mRNA levels measured by qPCR in HEK293T cells transiently transfected with Flag or Flag-TRIM71 (*n* = 5). HPRT1 housekeeping gene was used for normalization. (**B**) Repression of a luciferase reporter under the control of the full length CDKN1A 3′UTR in HEK293T cells transiently transfected with Flag or Flag-TRIM71 (*n* = 7). (**C**) Representative immunoblot showing levels of Ig-Ctrl/Ig-TRIM32/Ig-TRIM71/Ig-ΔNHL6 expression in total lysates (input) and pull down fractions (IP) after RNA-IP with Protein G Dynabeads. (**D**) CDKN1A mRNA IP enrichment and (**E**) HPRT1 mRNA IP enrichment after RNA-IP in HEK293T cells transiently transfected with Ig-Ctrl/Ig-TRIM32/Ig-TRIM71/Ig-ΔNHL6 (*n* = 4). Quantifications of specific mRNAs were performed by qPCR and IP enrichment was calculated as 2^∧^-((IP_target_-IP_ref_)-(INPUT_target_-INPUT_ref_)), where 18S rRNA was used as a reference (ref) for normalization. (**F**) Total RNA enrichment in Ig-Ctrl/Ig-TRIM32/Ig-TRIM71/Ig-ΔNHL6 RNA-IPs, calculated as Total RNA (IP)/Total RNA (INPUT) for each condition (n = 7–8). Quantifications of total RNA were based on the optical density (OD) at 260nm measured using a Nanodrop. (**G**) Repression of the full length CDKN1A 3′UTR luciferase reporter in HEK293T cells transiently transfected with Flag-Ctrl/Flag-TRIM32/Flag-TRIM71/Flag-ΔNHL6 (*n* = 3–4). Norm. RLU = Normalized Relative Light Units. (**H**) Representative immunoblot showing the expression levels of the different constructs used in the luciferase assay depicted in G. All graphs represent Mean±SEM.

To further characterize TRIM71-mediated CDKN1A regulation, we transiently overexpressed TRIM71 in HEK293T cells. Again, TRIM71 overexpression led to reduced CDKN1A mRNA levels (Figure 2A). TRIM71 has been reported to mediate mRNA silencing in *C. elegans* via either translational inhibition or mRNA degradation through 5′UTR or 3′UTR recognition, respectively (31). In mES cells, TRIM71-mediated Cdkn1a mRNA repression occurred via 3′UTR recognition (25). In line with the observed mRNA downregulation, overexpression of TRIM71 in HEK293T cells resulted in the repression of a luciferase reporter under the control of the full length human CDKN1A 3′UTR (Figure 2B). We then investigated whether TRIM71 was able to physically interact with CDKN1A mRNA by conducting UV-crosslinking RNA immunoprecipitations (RNA-IPs) in HEK293T overexpressing Ig-Ctrl, Ig-TRIM71, Ig-TRIM32, another member of the TRIM-NHL protein family, and Ig-ΔNHL6 (Figure 2C), a TRIM71 mutant which phenocopied the total loss of TRIM71 expression during murine development (19). TRIM71 pull down fractions showed a specific CDKN1A mRNA enrichment of 20-fold over the Ig-Ctrl (Figure 2D). A mild, unspecific HPRT1 enrichment was observed for both TRIM71 and TRIM32 pull down fractions (Figure 2E), and these two constructs also pulled down comparable amounts of total RNA (Figure 2F), highlighting the target specificity of TRIM71 for CDKN1A mRNA. Interestingly, the deletion of the last NHL repeat totally abrogated mRNA binding (Figure 2D–F). Consistent with the CDKN1A mRNA binding ability of the different constructs, only TRIM71 —but neither TRIM32 nor ΔNHL6—was able to repress the CDKN1A 3′UTR (Figure 2G) upon overexpression in HEK293T cells (Figure 2H).

**Figure 3.**
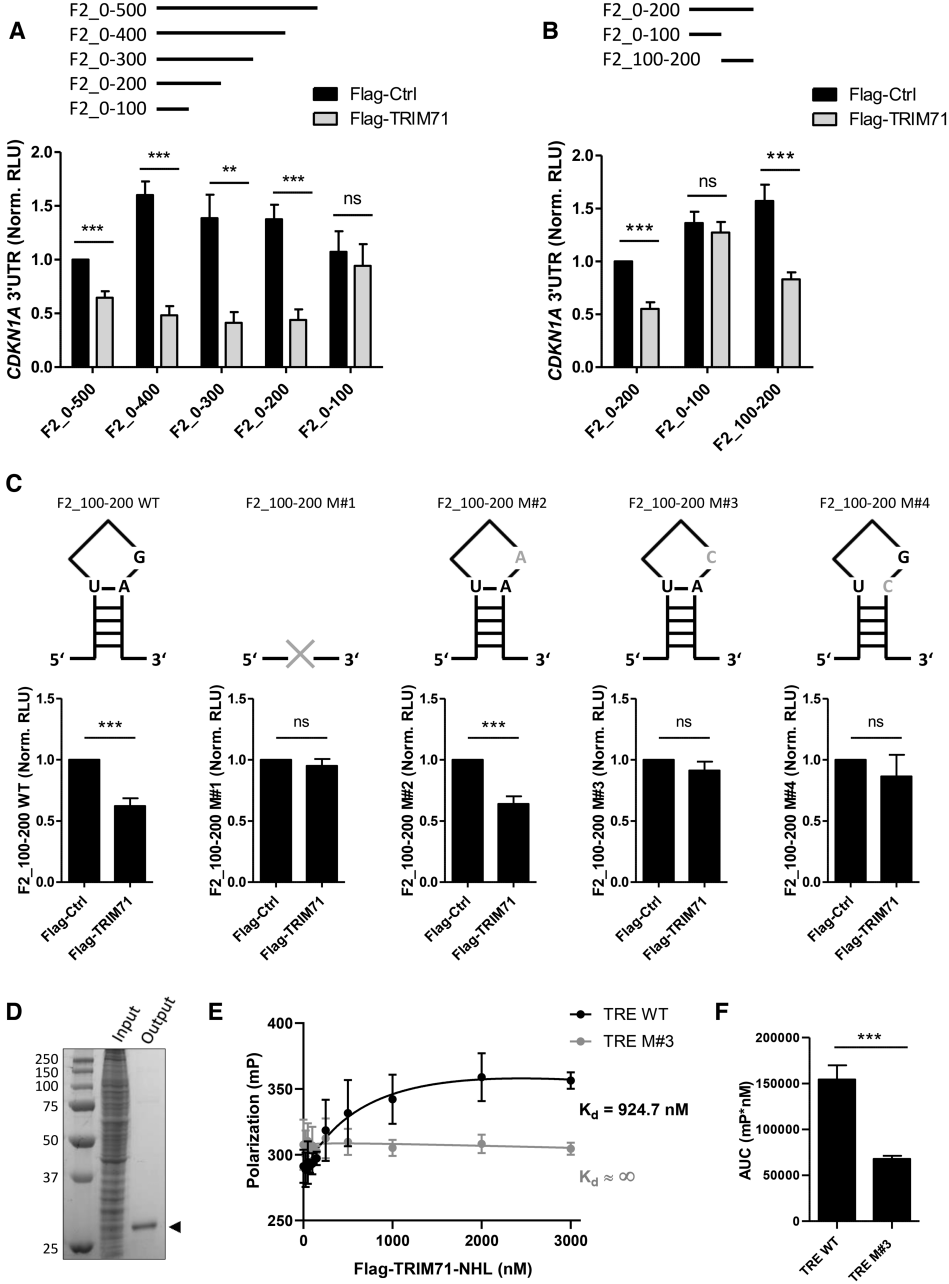
TRIM71 directly interacts with a structural RNA motif present in the 3′UTR of CDKN1A mRNA. (**A**) Repression of a luciferase reporter under the control of different CDKN1A 3′UTR F2 fragments (depicted above the graph) in HEK293T cells transiently transfected with Flag or Flag-TRIM71. F2_0–500 (510bp) = F2 used in Supplementary Figure 5B and C, spanning from 508–1017 bp within the full length 3′UTR. F2_0–400 (401bp), resulting from a 109bp deletion from the 3′ end of F2_0–500. F2_0–300 (292 bp), resulting from a 218 bp deletion from the 3′ end of F2_0–500. F2_0–200 (214 bp), resulting from a 296bp deletion from the 3′ end of F2_0–500. F2_0–100 (106 bp), resulting from a 405 bp deletion from the 3′ end of F2_0–500 (*n* = 4). (**B**) Repression of a luciferase reporter under the control of different CDKN1A 3′UTR F2 fragments, namely F2_0–200, F2_0–100 (both used in A) and F2_100–200 (*n* = 4–5). (**C**) Repression of a luciferase reporter under the control of the CDKN1A 3′UTR fragment F2_100–200, in which the stem-3base-loop (SL) structure has been mutated as depicted by the schematic drawings on top of each corresponding graph: Mutant 1 (M#1) = 13-mer SL deleted; Mutant 2 (M#2) = permissive G/A mutation; Mutant 3 (M#3) = disruptive G/C mutation; Mutant 4 (M#4) = disruptive A/C mutation. (*n* = 3). Norm. RLU = Normalized Relative Light Units. (**D**) Coomassie Staining of a 12% PAGE-SDS gel loaded with input (total lysates) and output (α-Flag IP fraction) protein lysates of CV1 cells expressing human Flag-TRIM71-NHL recombinant protein (33 kDa). Details on protein expression and native protein purification can be found in Supplementary Methods. (**E**) Fluorescence Polarization Assays showing binding-saturation curves and equilibrium dissociation constants (*K*_d_) for the interaction between purified human Flag-TRIM71-NHL recombinant protein and two synthetized 13-mer ssRNAs, namely TRE WT and TRE M#3 (*n* = 3). mP = units of millipolarization. (**F**) Area under the Curve (AUC) calculated for the binding-saturation curves of individual experiments (*n* = 3) collectively depicted in E. See also Supplementary Figure S5. All graphs represent Mean±SEM.

A previous study in mES cells proposed TRIM71-mediated mRNA repression to be assisted by miRNAs given that TRIM71 repressed a fragment of the mouse Cdkn1a 3′UTR in cooperation with the ES-specific miR-302 (25). However, the human CDKN1A 3′UTR lacks such a miRNA binding site (Supplementary Figure S5A). This prompted us to identify the responsive element(s) for TRIM71 in the human CDKN1A 3′UTR. To this end, we cloned the 3′UTR of CDKN1A in three consecutive 500 bp fragments and evaluated TRIM71-mediated repression of these fragments compared to the repression of the full length 3′UTR (FL) by luciferase assays in HEK293T cells (Supplementary Figure S5B–D). TRIM71-mediated repression was observed in all three fragments, indicating that several TRIM71 responsive elements are distributed along the whole CDKN1A 3′UTR (Supplementary Figure S5B). However, F2 was the most strongly repressed fragment, while the repression ratios for F1 and F3 were significantly lower (Supplementary Figure S5C) although TRIM71 expression levels were similar (Supplementary Figure S5D). We therefore performed serial deletions of 100 bp in the 3′end of F2, resulting in the construction of 4 reporters (F2_0–400, F2_0–300, F2_0–200 and F2_0–100), and compared their repression to that on full F2 (now called F2_0–500). TRIM71 significantly repressed the luciferase activity of F2_0–400, F2_0–300 and F2_0–200 reporters, while repression was completely abrogated for the F2_0–100 reporter despite strong TRIM71 overexpression (Figure 3A and Supplementary Figure S5E). Since these results indicated that a recognition site for TRIM71 is located between 100–200 bp within the F2 fragment, we then cloned a reporter containing only the F2_100–200 region and confirmed TRIM71-mediated repression of this fragment (Figure 3B and Supplementary Figure S5E). Recently, the *C. elegans* LIN-41 (CeLIN41) responsive element (LRE) has been identified as an RNA structural motif consisting of a stem/3-nucleotide-loop (SL) with specific nucleotides in conserved positions (Supplementary Figure S5F) (32). Thus, we used the sequence of F2_100–200 for *in silico* RNA secondary structure prediction and identified a candidate SL structure (with the nucleotide sequence GUCUUGUGAAGGC) in this region (Supplementary Figure S5G-H). Subsequently, we cloned several F2_100–200 reporters harboring distinct mutations of that prospective RNA SL motif and evaluated their repression by TRIM71 (Figure 3C). Deletion of the full SL structure from F2_100–200 (M#1 ΔSL) or mutations in the conserved positions reported to disrupt the SL structure of the LRE (32) (M#3 G/C and M#4 A/C) completely abrogated TRIM71-mediated repression of F2_100–200, whereas a predicted permissive mutation (32) (M#2 G/A) showed a repression comparable to the wild type (WT) F2_100–200 (Figure 3C). Therefore, we now term this RNA SL motif *TRIM71 responsive element* (TRE).

The NHL domains of TRIM71 orthologues Brat (*D. melanogaster*) and CeLIN41 were shown to mediate direct RNA interaction (31,33,34), and recently, Kumari *et al.*, demonstrated the direct interaction of the FLN-NHL domains of CeLIN41 and *D. rerio* Lin41 (DrLIN41) with their reported LRE (32). The sequences from zebrafish and human share an overall identity of 84% over the 390 amino acids of these domains, and a sequence similarity of 96% in this region (data not shown). Based on these similarities, we were able to model the human TRIM71 FLN-NHL domains complexed with the TRE present in the CDKN1A 3′UTR F2_100–200 fragment (Supplementary Figure S5J and K). Importantly, all 14 residues identified in DrLIN41 NHL domain which interacted with the LRE are conserved in the human TRIM71 protein. Specifically, the positively-charged binding pocket formed by the β-propeller structure of the NHL domain comprises the residues R608, R625, R655, K672, R720, R751, R796 and R814, and is maintained in the human TRIM71 NHL domain. These residues are predicted to form electrostatic interactions with the negatively charged phosphate groups of the 3-mer RNA loop section (I–III) and the succeeding A (+1) nucleotide of the first U-A stem pair (Supplementary Figure S5J and K).

To confirm direct TRIM71 binding to the TRE that we had identified in the CDKN1A 3′UTR F2_100–200 fragment, we purified the human Flag-tagged TRIM71 NHL domain (without including the FLN domain) via recombinant vaccinia virus-driven protein production in CV1 cells (Figure 3D). We then performed fluorescence polarization experiments using two different 5′Cy3-labelled 13-mer ssRNAs (TRE WT and TRE M#3 (Supplementary Figure S5I)) and increasing Flag-TRIM71-NHL concentrations. Consistent with our luciferase assays, which showed that TRIM71-mediated repression was abrogated by the G/C (III) mutation (M#3), the human TRIM71 NHL domain robustly bound the TRE WT RNA but not the TRE M#3 RNA (Figure 3E and F). The affinity of the TRIM71 NHL domain for our TRE WT 13-mer stem loop U(-1)-G(I)-U(II)-G(III)-A(+1) (*K*_d_: 0.925 μM) is comparable to the previously published binding constant of the CeLIN41 for the LRE 13-mer stem loop U(-1)-C(I)-C(II)-A(III)-A(+1) (*K*_d_: 1.322 μM) (32).

Altogether, our findings show that TRIM71 is able to physically and specifically interact with CDKN1A mRNA to mediate its repression via 3′UTR recognition followed by mRNA degradation. Furthermore, we demonstrate that the TRIM71 NHL domain is sufficient to mediate a direct interaction with the TRE that we identified in the 3′UTR of the CDKN1A mRNA.

### TRIM71-mediated CDKN1A 3′UTR repression correlates with P-body localization

**Figure 4.**
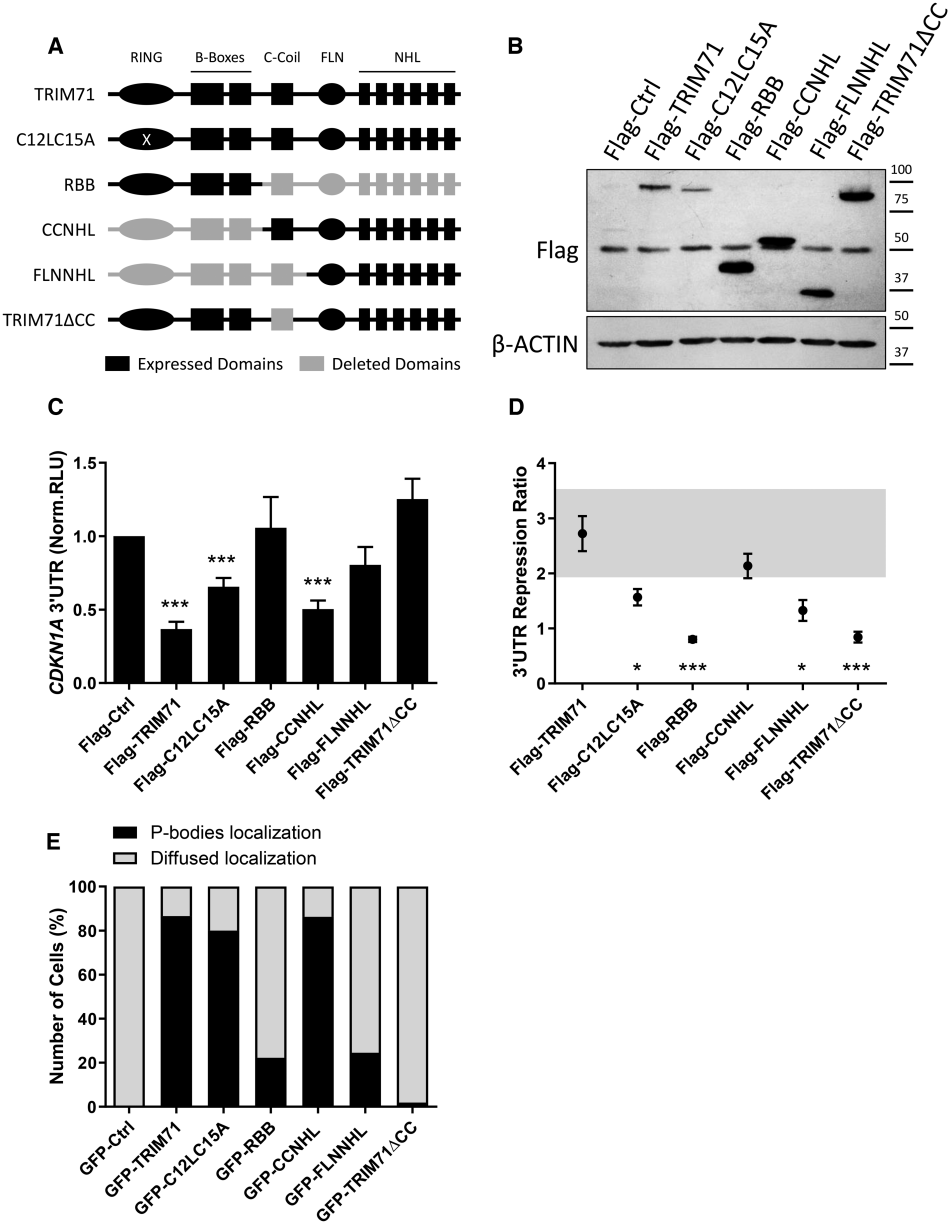
TRIM71-mediated CDKN1A 3′UTR repression correlates with P-body localization. (**A**) Representation of TRIM71 constructs used in B–E. (**B**) Representative immunoblot showing the expression levels of the different truncated TRIM71 constructs (depicted in A) used in the luciferase assay depicted in C-D. (**C**) Repression of the full length CDKN1A 3′UTR luciferase reporter in HEK293T cells transiently transfected with different Flag-tagged TRIM71 constructs from A–B (*n* = 4–7). Norm. RLU = Normalized Relative Light Units. (**D**) Repression Ratio representing the capability of each Flag-tagged TRIM71 construct to repress the full length CDKN1A 3′UTR luciferase reporter, calculated from values shown in B as: Repression Ratio _Flag-Construct X_ = norm.RLU_Flag-empty_/norm.RLU_Flag-Construct X_. The grey shaded area represents the SD of the Flag-TRIM71 full length wild type construct. (**E**) Percentage of HEK293T cells in which the overexpressed GFP-tagged constructs colocalized with the P-body specific marker DCP1A (coexpressed in HEK293T RFP-tagged). A total of at least 50 cells per condition were observed for GFP-RFP colocalization under a confocal laser scanning microscope (see also Supplementary Figure S6). All graphs represent mean ± SEM.

To elucidate which other domains may be important for 3′UTR repression, we cloned several truncated TRIM71 constructs (Figure 4A) and performed luciferase reporter assays by overexpressing them in HEK293T cells together with our full length CDKN1A 3′UTR reporter (Figure 4B–D). Mutations impairing TRIM71 E3 ligase activity (C12LC15A) (18,27,35) did not abrogate TRIM71-mediated CDKN1A 3′UTR repression (Figure 4C), indicating that TRIM71-mediated ubiquitylation is not required for mRNA regulation, in line with previous studies showing that TRIM71 RING domain is dispensable for such a function (27). However, the repression strength was strikingly reduced in this mutant (Figure 4D). Such a reduction may be explained by lower expression levels of C12LC15A compared to those of full-length wild type TRIM71 (Figure 4B), although we cannot rule out that ubiquitylation-dependent events might be involved in fine-tuning or optimization of mRNA repression. A construct consisting of the RING domain and the two B-Boxes (RBB) failed to repress CDKN1A 3′UTR, while a construct lacking such domains (CCNHL) retained its repression ability. Interestingly, deletion of the CC region from that construct (FLN-NHL) abrogated CDKN1A 3′UTR repression, demonstrating that the NHL domain is required but not sufficient for 3′UTR repression, since the CC region also seems to be essential. We then deleted the CC from a full-length construct (TRIM71ΔCC) and confirmed that CC-lacking TRIM71 was no longer able to repress the 3′UTR of CDKN1A (Figure 4B-D).

Several previous studies have shown that truncated versions of TRIM71 were unable to locate to P-bodies (18,25). Hence, we studied whether the capability of TRIM71 to repress CDKN1A was connected to its P-body localization. To do so, we overexpressed several TRIM71 constructs (Figure 4A) together with the P-body specific marker DCP1A (36) and evaluated their colocalization (Figure 4E and Supplementary Figure S6). Only protein constructs containing the CC region were able to locate to P-body structures, while all constructs lacking the CC region, which failed to repress CDKN1A 3′UTR in our reporter assays, were also unable to localize to P-bodies. Instead, they were evenly distributed through the cellular cytoplasm and in some cases even entered the nuclei (Supplementary Figure S6). Altogether, we show that TRIM71 CC region is required for P-body localization and CDKN1A 3′UTR repression. Our data reveals a correlation between the ability of different TRIM71 constructs to induce 3′UTR repression and their proper localization within P-bodies, suggesting that TRIM71-mediated mRNA regulation may occur in such organelles.

### TRIM71-mediated CDKN1A mRNA repression is miRNA-independent

**Figure 5.**
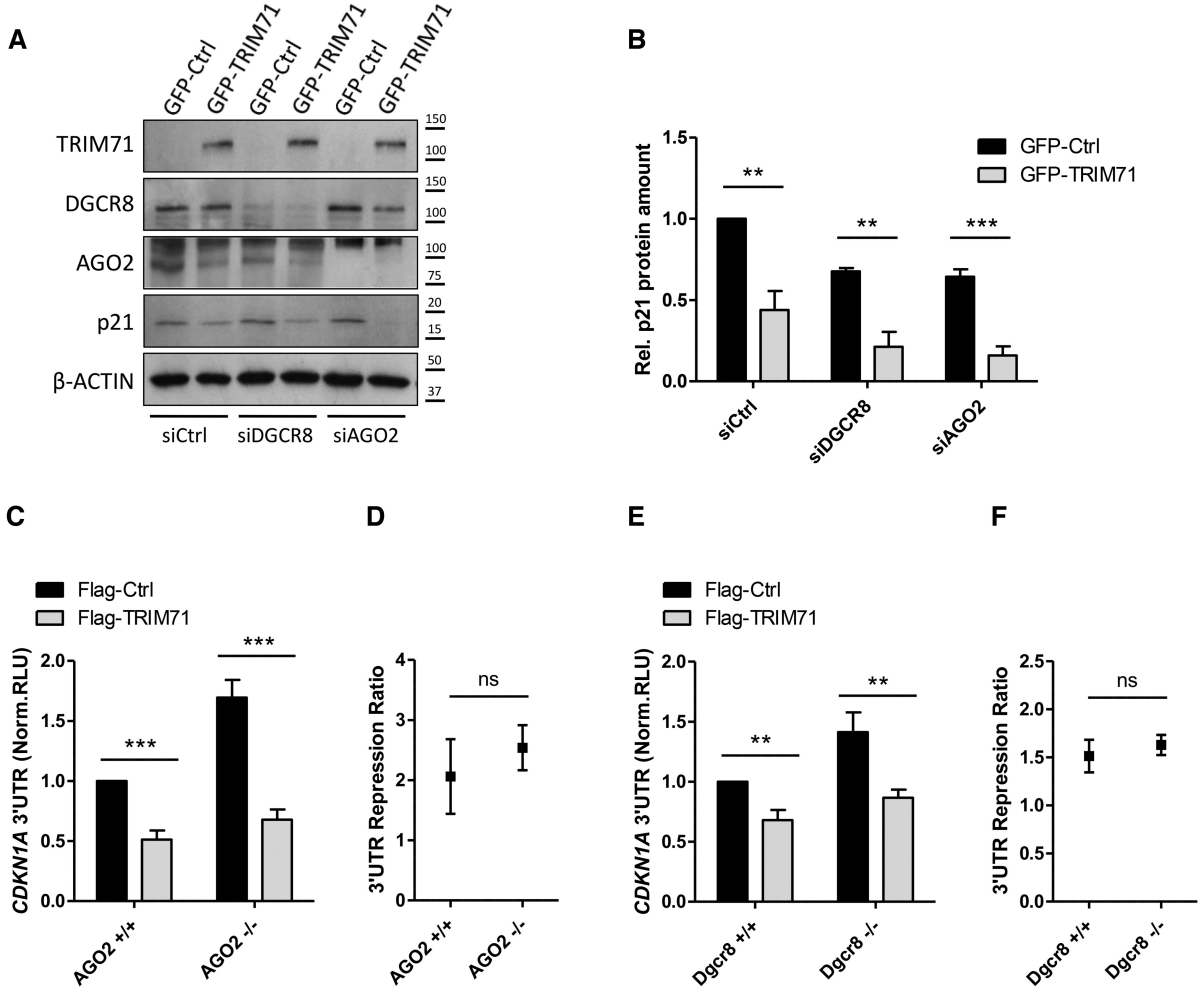
TRIM71-mediated CDKN1A mRNA repression is miRNA-independent. (**A**) Representative immunoblot showing p21 protein levels in HEK293 cells stably overexpressing GFP or GFP-TRIM71 upon DGCR8 or AGO2 knockdown. (**B**) Quantification of p21 band densitometry relative to ACTIN bands from replicate blots of A (*n* = 3). (**C**) Repression of the full length CDKN1A 3′UTR luciferase reporter in wild type (AGO2 +/+) or AGO2 knockout (AGO2 –/–) HEK293T cells transiently transfected with Flag or Flag-TRIM71 (*n* = 3). (**D**) Repression ratio calculated from data depicted in C, as specified in Figure 4D. (**E**) Repression of the full length CDKN1A 3′UTR luciferase reporter in wild type (Dgcr8+/+) or Dgcr8 knockout (Dgcr8–/–) mES cells transiently transfected with Flag or Flag-TRIM71 (*n* = 3). (**F**) Repression ratio calculated from data depicted in E, as specified in Figure 4D (see also Supplementary Figure S7). Norm. RLU = Normalized Relative Light Units. All graphs represent mean ± SEM.

P-bodies are organelles for RNA surveillance where several RNA degradation pathways occur (36), including the miRNA-mediated mRNA silencing (37). In mES cells, TRIM71-mediated Cdkn1a 3′UTR repression was assisted by miR-302 (25), whereas in human cells TRIM71 was shown to repress several mRNAs without the assistance of either AGO2 or miRNAs (27). Since no conserved miRNA binding sites were found within the F2_100–200 sequence (see again Supplementary Figure S5A), we next investigated whether TRIM71 could also repress the full length CDKN1A 3′UTR independently from the miRNA machinery. We therefore interfered with miRNA expression and activity by knocking down DGCR8 and AGO2 respectively in HEK293T cells. CDKN1A/p21 protein remained downregulated by TRIM71 upon DGCR8 and AGO2 knockdowns (Figure 5A and B), suggesting that TRIM71 can regulate CDKN1A without miRNA assistance. Further confirming AGO2 independency of this mechanism, overexpressed TRIM71 continued to repress CDKN1A 3′UTR in AGO2 knockout HEK293T cells (Figure 5C, D and Supplementary Figure S7A). However, overexpression of let-7 miRNA mimic in AGO2 knockout HEK293T cells resulted in the repression of its *bona fide* target HMGA2 (Supplementary Figure S7B), showing that these cells were still able to conduct miRNA-mediated silencing and suggesting that other AGO proteins may take over this role. Therefore, in order to confirm miRNA independence of TRIM71-mediated CDKN1A 3′UTR repression, we used Dgcr8 knockout mES cells (Supplementary Figure S7C), which are unable to produce mature functional miRNAs (25) (Supplementary Figure S7D). TRIM71 overexpression resulted in CDKN1A 3′UTR repression despite the absence of miRNAs (Figure 5E-F). Conversely, TRIM71 knockdown (Supplementary Figure S7E) relieved the repression of the CDKN1A 3′UTR reporter in both WT (Supplementary Figure S7F) and Dgcr8 knockout (Supplementary Figure S7G) mES cells. Altogether, our findings demonstrate that TRIM71-mediated CDKN1A 3′UTR repression can occur in an AGO2- and a miRNA-independent manner.

### TRIM71 and NMD components cooperate to repress CDKN1A mRNA

**Figure 6.**
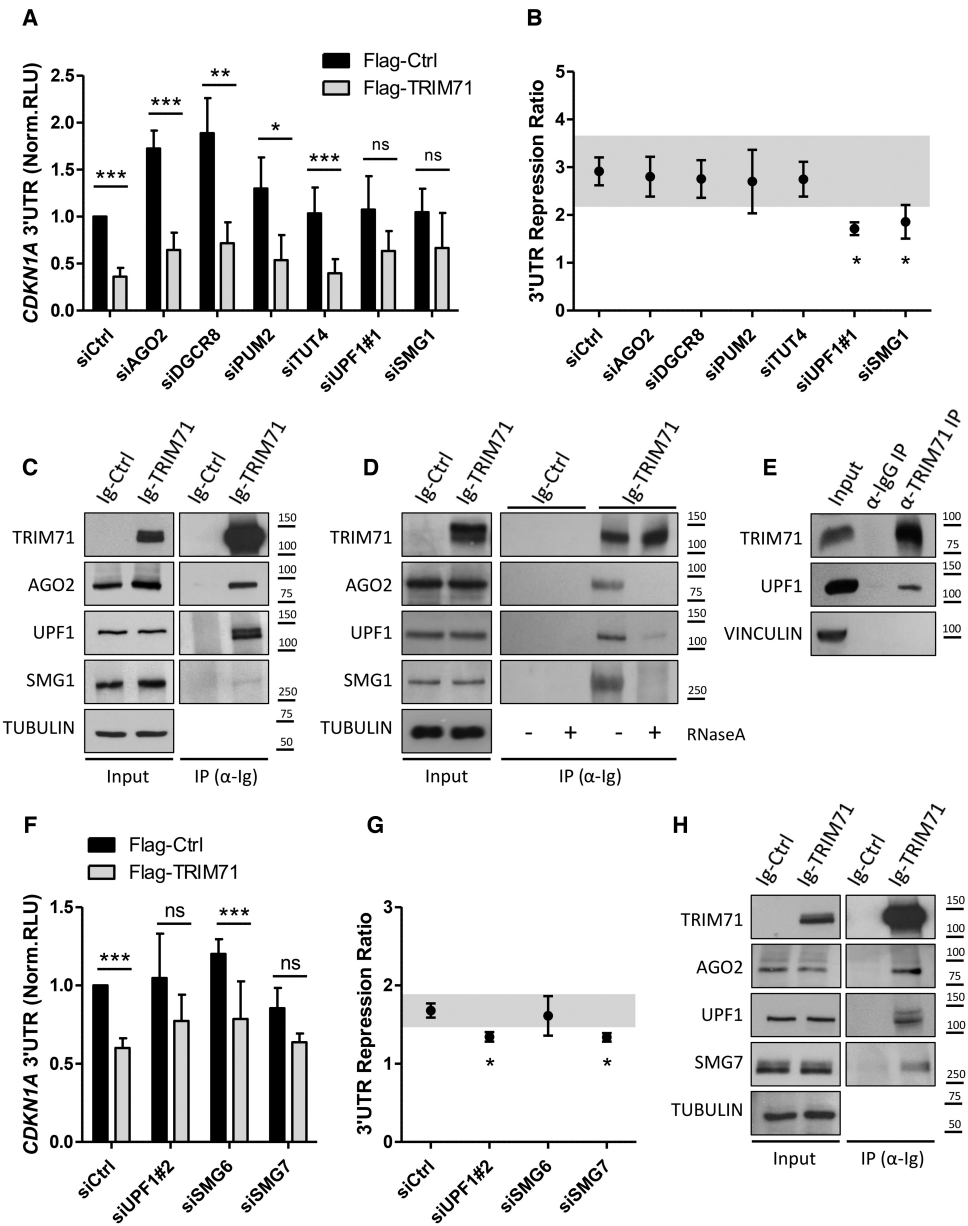
TRIM71 relies on NMD components to repress CDKN1A 3′UTR. (**A**) Repression of the full length CDKN1A 3′UTR luciferase reporter in HEK293T cells transiently transfected with Flag or Flag-TRIM71 upon knockdown of the indicated P-body proteins (*n* = 3–6). (**B**) Repression ratio calculated from data depicted in A, as specified in Figure 4D. (**C**) Representative immunoblot showing UPF1, SMG1 and AGO2 coprecipitation with Ig-TRIM71 in HEK293T cells. (**D**) Representative immunoblot showing UPF1, SMG1 and AGO2 interaction with Ig-TRIM71 in HEK293T cells upon RNase A treatment. (**E**) Immunoblot showing the coprecipitation of UPF1 with endogenous TRIM71 in HepG2 cells. (**F**) Repression of the full length CDKN1A 3′UTR luciferase reporter in HEK293T cells transiently transfected with Flag or Flag-TRIM71 upon knockdown of the indicated NMD proteins (*n* = 3–4). (**G**) Repression ratio calculated from data depicted in F, as specified in Figure 4D. (**H**) Representative immunoblot showing UPF1, SMG7 and AGO2 coprecipitation with Ig-TRIM71 in HEK293T cells. (see also Supplementary Figure S8). Norm. RLU = Normalized Relative Light Units. Graphs A and F represent Mean±SD, while for B and F, grey shaded areas represent the SD of the siCtrl sample and error bars represent the SEM.

Since our data show that TRIM71 represses CDKN1A mRNA without relying on the miRNA pathway, we investigated which other pathways may assist TRIM71 in mRNA repression. To this end, we evaluated TRIM71-mediated full length CDKN1A 3′UTR repression (Figure 6A and B) upon knockdown of several proteins (Supplementary Figure S8A and B) which are also present in P-bodies and linked to mRNA degradation. We included AGO2 and DGCR8 again as a control, the RNA-binding protein PUM2, known to be a TRIM71 binding partner and to share mRNA targets with TRIM71 (27), the TUT4 enzyme, responsible for targeting specific miRNAs and mRNAs for degradation via uridylation (38,39), and UPF1 and SMG1, two effectors of the NMD pathway (40). Consistent with our previous data, AGO2 and DGCR8 knockdowns did not affect TRIM71 ability to repress CDKN1A 3′UTR in HEK293T cells. Similarly, the knockdowns of PUM2 or TUT4 had no effect on TRIM71-mediated CDKN1A 3′UTR repression. Of note, a previous study revealed that PUM motifs were not enriched in TRIM71 mRNA targets and that PUM knockdown did not affect TRIM71-mediated mRNA repression (27), as we have confirmed. Interestingly, impairment of NMD by either UPF1 or SMG1 knockdown decreased the capability of TRIM71 to repress CDKN1A 3′UTR (Figure 6A and B), suggesting that these proteins cooperate with TRIM71 in this function. Protein immunoprecipitation demonstrated coprecipitation of endogenous UPF1 and SMG1 with ectopically expressed TRIM71 in HEK293T cells (Figure 6C and D). Moreover, UPF1 also coprecipitated with endogenous TRIM71 in HepG2 cells (Figure 6E). TRIM71–UPF1 interaction did not depend on TRIM71 E3 ligase activity, since UPF1 also coprecipitated with the C12LC15A mutant in HEK293T (Supplementary Figure S8C) and mES cells (Supplementary Figure S8D). Interestingly, our TRIM71 mutant ΔNHL6, which had failed to bind RNA (see again Figure 2D–F), could also not bind UPF1 (Supplementary Figure S8E), suggesting that the TRIM71–UPF1 interaction is RNA-dependent. Indeed, TRIM71 interactions with UPF1 and SMG1 were disrupted after RNase treatment (Figure 6D), implying that TRIM71, UPF1 and SMG1 bind to common mRNA targets.

**Figure 7.**
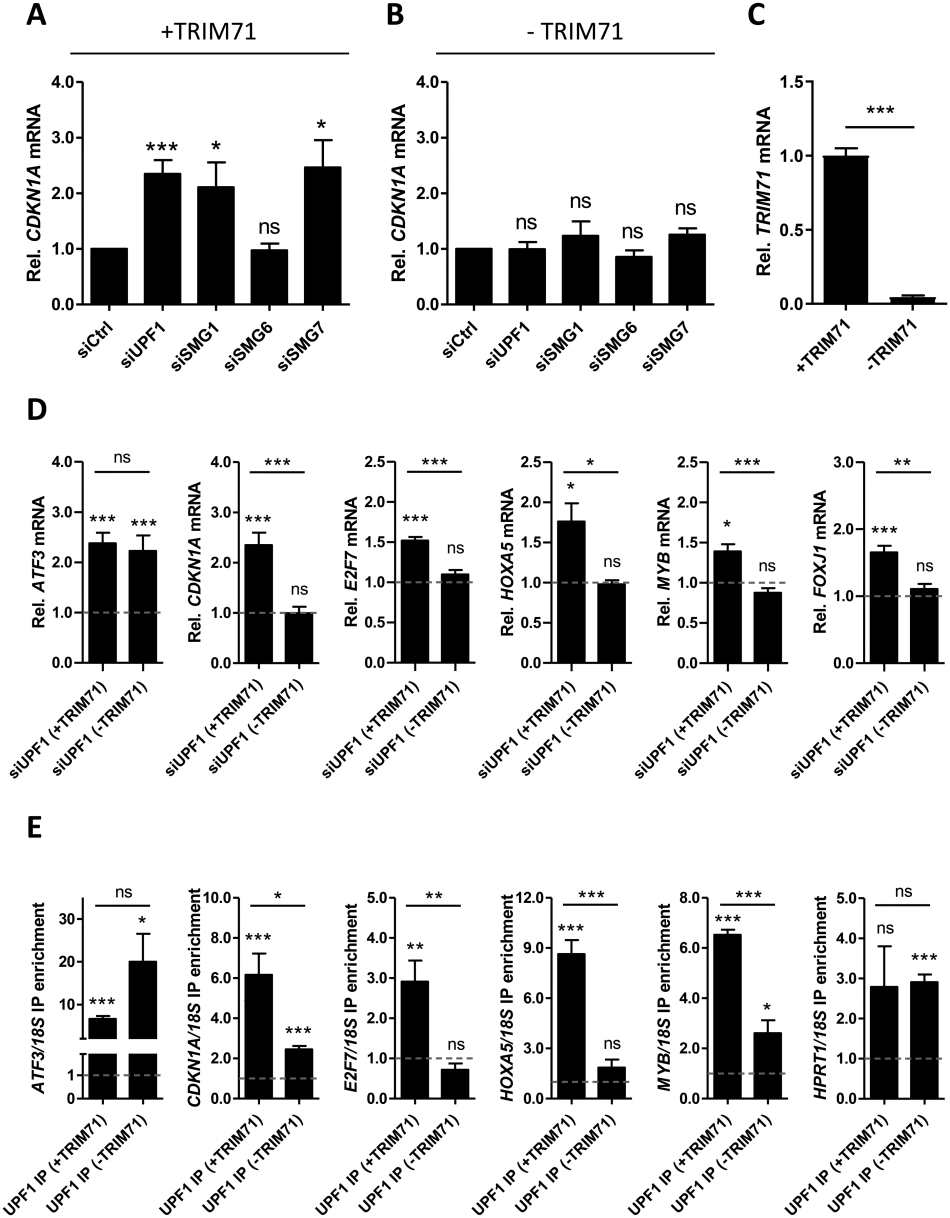
TRIM71 and NMD components cooperate in the repression of specific target mRNAs. (**A**) qPCR quantification of endogenous CDKN1A mRNA levels upon knockdown of NMD components in the presence of TRIM71 (+TRIM71) (*n* = 4) or (**B**) its absence (–TRIM71) (*n* = 4), achieved by TRIM71 knockdown in HEK293T cells, which endogenously express low TRIM71 levels. (**C**) qPCR quantification of TRIM71 levels corresponding to presence (+) and absence (–) of TRIM71 from A–E (*n* = 3). (**D**) qPCR quantification of several mRNA targets upon UPF1 knockdown (siUPF1) in HEK293T cells (compared to siCtrl cells, represented by the line at y = 1) in the presence (+) and absence (–) of TRIM71 (*n* = 3–6). HPRT1 housekeeping gene was used for normalization. (**E**) IP enrichment of several mRNA targets after RNA-IP with Flag-UPF1 (UPF1 IP) overexpressed in HEK293T cells (compared to Flag-Ctrl IP, represented by the line at *y* = 1) in the presence (+) and absence (-) of TRIM71. IP enrichment was calculated as 2 ((IPtarget – IPref) – (INPUTtarget – INPUTref)), where 18S rRNA was used as a reference (ref) for normalization, whereas HPRT1 was used in this case as a non-target (*n* = 3–4). All graphs represent mean ± SEM.

Once SMG1 and UPF1 initiate NMD, the degradation of the transcript is triggered by the recruitment of SMG6 and/or SMG5-SMG7 dimers (41). Therefore, we investigated TRIM71-mediated CDKN1A 3′UTR repression upon SMG6 and SMG7 knockdowns (Supplementary Figure S8A and B) and found that SMG7 knockdown, but not SMG6 knockdown, decreased TRIM71-mediated CDKN1A 3′UTR repression (Figure 6F and G). Endogenous SMG7 coprecipitated with overexpressed TRIM71 in HEK293T cells (Figure 6H), and conversely, endogenous TRIM71, together with UPF1 and SMG1, coprecipitated with endogenous SMG7 in HepG2 cells (Supplementary Figure S8F). Altogether, our data suggest that TRIM71-mediated CDKN1A 3′UTR repression results from SMG1/UPF1/SMG7-mediated decay. To confirm this, we measured endogenous CDKN1A mRNA levels upon NMD components knockdown in HEK293T cells. In line with our luciferase assay results, CDKN1A mRNA was upregulated upon UPF1, SMG1 and SMG7 knockdowns, but not SMG6 knockdown (Figure 7A). More importantly, this upregulation occurred only in the presence of TRIM71 (+TRIM71), whereas the knockdown of NMD components in the absence of TRIM71 (-TRIM71) had no effect on CDKN1A endogenous mRNA levels (Figure 7B and C), demonstrating that CDKN1A mRNA regulation by NMD is TRIM71-dependent. Of note, the knockdown of SMG6 was not efficient enough (40%) to completely exclude a participation of SMG6 in CDKN1A regulation.

Although TRIM71 and NMD factors are post-transcriptional mRNA regulators, a transcriptional contribution could account for the observed CDKN1A mRNA upregulation. In fact, TRIM71 has been shown to destabilize p53 protein in mES cells undergoing differentiation (24), and p53 is a well-known CDKN1A/p21 transcriptional activator. Furthermore, p53 is stabilized and activated (phosphorylated) upon DNA damage, and NMD components are involved in DNA damage response (42–45). In order to account for a possible upregulation of CDKN1A mRNA resulting from p53-mediated transcriptional activation upon the knockdown of TRIM71 and/or NMD components, we investigated DNA damage levels via phospho-H2AX (Ser139) staining, and p53/phospho-p53 (Ser15) levels via western blot analysis in HEK293T cells under such conditions (Supplementary Figure S9). Neither DNA damage levels (Supplementary Figure S9A and B) nor total p53 levels (Supplementary Figure S9C and D) were altered upon knockdown of TRIM71 or NMD components, as compared to siCtrl cells. Furthermore, phospho-p53 was undetected under these conditions (Supplementary Figure S9C), being only upregulated upon exposure to UV-radiation, which served as a positive control for DNA damage (Supplementary Figure S9). These findings exclude the possibility that CDKN1A mRNA upregulation results from p53-dependent transcriptional activation in our experimental conditions.

Our luciferase reporter assays showed that TRIM71 relies on NMD factors for the repression of CDKN1A 3′UTR (Figure 6), and conversely, the measurements of endogenous CDKN1A mRNA levels showed that NMD impairment only led to CDKN1A upregulation in the presence of TRIM71 (Figure 7A–C). These data demonstrate that TRIM71 and NMD cooperate in an interdependent manner to regulate CDKN1A and prompted us to investigate whether other known NMD targets and TRIM71 targets were also regulated by the TRIM71/NMD axis that we have identified. Interestingly, the known TRIM71 targets E2F7, HOXA5, MYB and FOXJ1 (27,28) were also upregulated upon UPF1 knockdown in the presence of TRIM71 but not in its absence, while the canonical (EJC-dependent) NMD PTC-containing targets ATF3, TBL2, GADD45A and GADD45B (46–49) were derepressed upon UPF1 knockdown regardless of the presence of TRIM71 (Figure 7D and Supplementary Figure S10A). Consistent with these results, CDKN1A, E2F7, HOXA5 and MYB mRNAs coprecipitated with overexpressed UPF1 in HEK293T cells only in the presence of TRIM71, while UPF1 interaction with those mRNAs was diminished or abrogated upon TRIM71 depletion (Figure 7E). The specific interaction of UPF1 with its canonical targets ATF3 and GADD45A was observed in both the presence and absence of TRIM71, as well as its unspecific interaction with the housekeeping HPRT1 mRNA (Figure 7E and Supplementary Figure S10B). These results clearly show that TRIM71 enables UPF1 recruitment and UPF1-dependent decay of specific PTC-lacking mRNAs, while it appears dispensable for the degradation of canonical NMD targets. Supporting this notion, endogenous TRIM71 expression was not altered upon knockdown of NMD components (Supplementary Figure S10C) while NMD components were found to cross-regulate each other (Supplementary Figure S10D–F), as previously described (50).

**Figure 8.**
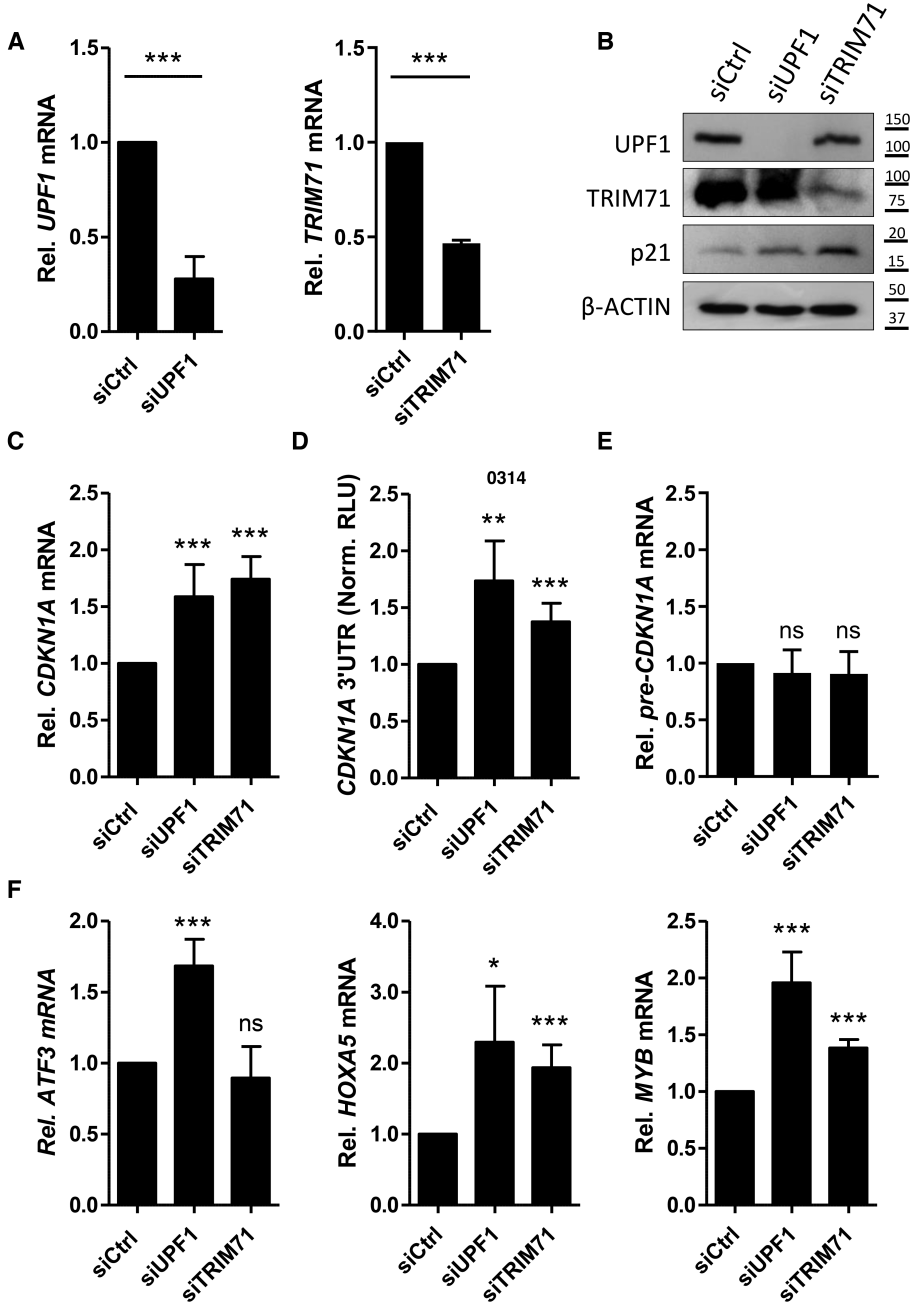
CDKN1A mRNA is post-transcriptionally regulated by TRIM71 and UPF1 in hepatocellular carcinoma. (**A**) Levels of UPF1 knockdown (siUPF1) and TRIM71 knockdown (siTRIM71) reached in HepG2 cells (*n* = 4). (**B**) Immunoblot showing the upregulation of CDKN1A/p21 protein levels upon knockdown of UPF1 and TRIM71 in HepG2 cells. (**C**) Endogenous CDKN1A mRNA levels measured by qPCR in HepG2 cells upon knockdown of UPF1 and TRIM71 (*n* = 5–8). (**D**) Repression of the full length CDKN1A 3′UTR luciferase reporter in HepG2 cells upon knockdown of UPF1 and TRIM71 (*n* = 3–4). (**E**) Endogenous CDKN1A pre-mRNA levels measured by qPCR in HepG2 cells upon knockdown of UPF1 and TRIM71 (*n* = 3–6). (**F**) mRNA levels of the indicated targets measured by qPCR in HepG2 cells upon knockdown of UPF1 and TRIM71 (*n* = 3–6). HPRT1 housekeeping gene was used for normalization. All graphs represent Mean±SD.

In order to evaluate whether CDKN1A mRNA was similarly regulated in hepatocellular carcinoma cells, we knocked down UPF1 in HepG2 cells (Figure 8A) and found upregulated p21 protein levels (Figure 8B) and CDKN1A mRNA levels (Figure 8C). Interestingly, according to the R2 genomic and visualization platform, UPF1 and CDKN1A expression are negatively correlated in samples from patients with advanced-stage HCC (Supplementary Figure S11). Moreover, both UPF1 and TRIM71 knockdowns relieved CDKN1A 3′UTR repression (Figure 8D), confirming that CDKN1A regulation occurs through 3′UTR recognition and mRNA degradation in HepG2 cells as well. Of note, CDKN1A pre-mRNA levels were found unaltered upon both UPF1 and TRIM71 knockdowns (Figure 8E), excluding a transcriptional contribution to this regulation. To further confirm a post-transcriptional regulation of CDKN1A via mRNA decay, HepG2 cells were treated with the transcription inhibitor Actinomycin D and CDKN1A mRNA decay was monitored over time. The stability of CDKN1A mRNA was increased in UPF1 and TRIM71 knockdown HepG2 cells as compared to siCtrl cells (Supplementary Figure S12). Similarly, the mRNA of the TRIM71 targets HOXA5 and MYB were also stabilized under the same conditions, while only UPF1 knockdown, but not TRIM71 knockdown, enhanced the stability of the canonical NMD target ATF3 (Supplementary Figure S12). These results were consistent with the changes in endogenous mRNA levels observed for these targets upon UPF1 and TRIM71 knockdown in HepG2 cells (Figure 8F).

Last, we found other TRIM71 mRNA targets that were not upregulated—and even several that were downregulated—upon UPF1 knockdown in HEK293T cells (Supplementary Figure S13A), suggesting that TRIM71 can also rely on factors other than NMD components for its mRNA repression function. However, how TRIM71 ‘chooses’ a given repression mechanism for each of its targets still remains to be investigated. In this context, comprehensive transcriptomic studies will be required to better characterize targets that are specifically regulated by the TRIM71/NMD axis in contrast to ‘TRIM71-only’ targets and ‘NMD-only’ targets (Supplementary Figure S13B). Altogether, our work uncovers a novel role for TRIM71 in non-canonical NMD, specifically targeting PTC-lacking mRNAs for NMD/UPF1-mediated decay, and it sheds further light on the RNA recognition and repression mechanisms of this stem cell-/cancer-specific protein.

## DISCUSSION

### TRIM71 represses CDKN1A expression via 3′UTR recognition followed by mRNA degradation, and promotes cancer cell proliferation

A pathological TRIM71 upregulation has been observed in 50% of HCC patients and is correlated with advanced tumor stages and poor prognosis (29). TRIM71 promoted proliferation of several hepatocellular carcinoma (HCC) cell lines presumably via ubiquitylation-mediated AGO1/2 protein destabilization (29). Although such a mechanism was previously observed in HEK293T and EC cells (18), those findings could not be reproduced by later studies (22,25,27,28). Similarly, we did not observe TRIM71-dependent changes in AGO2 stability in any of the cell lines tested, which included HEK293T, NCCIT, TCam-2, mES cells and HepG2 cells. Furthermore, key regulators of the miRNA pathway, including DROSHA, DGCR8, AGO1 and AGO2 are frequently overexpressed—rather than downregulated—in HCC (51), and specifically AGO2 overexpression has been shown to promote HCC progression, tumor vascularization (52) and metastasis (53). Accordingly, we showed that TRIM71 and AGO2 expression are positively correlated in HCC patient samples and found a negative correlation between TRIM71 and CDKN1A expression. Similar to earlier findings in mES and EC cells (25), we showed that TRIM71 mediates CDKN1A mRNA regulation and controls proliferation of HEK293 and HepG2 cancer cells. Previous studies in *C. elegans* postulated that CeLIN41-mediated mRNA silencing can be mediated via either 5′UTR recognition followed by translational inhibition or via 3′UTR recognition followed by mRNA degradation (31). We showed that TRIM71 overexpression in HEK293T cells resulted in the repression of the full length CDKN1A 3′UTR reporter, and conversely, TRIM71 knockdown in HepG2 cells relieved CDKN1A 3′UTR repression. These results correlated with changes in endogenous CDKN1A mRNA levels induced by TRIM71 in these cell lines, indicating that CDKN1A regulation occurs through 3′UTR recognition and mRNA degradation. Furthermore, our results highlighted a correlation between the capacity of different TRIM71 constructs to induce 3′UTR repression and their proper localization in P-bodies, revealing the requirement of the CC region for this function and suggesting that TRIM71-mediated mRNA repression may occur in such organelles. One of the best-characterized RNA degradation pathways occurring in P-bodies is the miRNA pathway. A previous study proposed that the mRNA repressive function of TRIM71 is assisted by miRNAs (25), whereas a later study reported the miRNA-independent repression of specific targets (27). Using AGO2 and DGCR8 knockdown and knockout mouse and human systems, we demonstrated that TRIM71-mediated CDKN1A 3′UTR repression can also occur independently from AGO2 and miRNAs. Thus, the contribution of TRIM71 to miRNA-mediated silencing as well as the functional relationship between TRIM71 and AGO2 with regard to the control of stem cell fate and cancer cell proliferation remain unclear. Nonetheless our findings clearly show that TRIM71 neither controls proliferation of HEK293 and HepG2 cells via AGO2 degradation nor mediates CDKN1A 3′UTR repression through AGO2-mediated mechanisms.

### The NHL domain of TRIM71 directly binds to a structural RNA stem-loop motif within the 3′UTR of the CDKN1A mRNA

TRIM71 has been described as a mRNA (54) and miRNA (55) binding protein. Specific RNA binding brings TRIM71 in close proximity with other RBPs which may assist its function(s). Indeed, many protein interactions mediated by TRIM71 are RNA-dependent (18,27). Therefore, interaction with RNA (directly or indirectly) is an essential requirement for its mRNA repressive function. Our UV-crosslinking RNA IP experiments showed that TRIM71, but not the related protein TRIM32, is able to physically and specifically bind CDKN1A mRNA, while the deletion of its last NHL repeat (ΔNHL6) completely abrogated mRNA interaction. Consistently, only TRIM71, but neither TRIM32 nor ΔNHL6, repressed the CDKN1A 3′UTR. The NHL domain of ortholog proteins Brat (*D. melanogaster*), CeLIN41 and DrLIN41 mediate direct interaction to RNA (31–33). Our results show for the first time a direct interaction between the human TRIM71 NHL domain and its target RNA. We have identified a TRIM71 responsive element (TRE) in the human CDKN1A 3′UTR which is similar to the previously reported CeLIN41 responsive element (LRE) (32), and we furthermore showed that the human TRIM71 NHL domain is sufficient for TRE binding, in contrast to the previous studies in CeLIN41 and DrLIN41 which used a FLN-NHL construct (31,32). A 100 bp fragment within the CDKN1A 3′UTR containing the TRE (F2_100–200) was sufficient for TRIM71-mediated luciferase repression, and mutations reported to affect the LRE structure (32) abrogated both F2_100–200 reporter repression and TRE direct binding. Our results collectively show that an intact NHL domain is sufficient for direct RNA interaction and thereby required for 3′UTR repression. Interestingly, most of the loss of function mutations in in CeLIN41 clustered within the NHL domain (16), and a mouse model which merely lacked the last NHL repeat (equivalent to our human ΔNHL6 mutant) phenocopied the total loss of TRIM71 (19 ), linking the mRNA repression role of TRIM71 to its essential functions during development. Furthermore, two recurrent TRIM71 *de novo* mutations located in the NHL domain (R608H and R796H), which were also found to disrupt RNA binding (56), have been recently identified in a cohort of Congenital Hydrocephalus patients (57). These findings have thus uncovered a novel role for TRIM71 in a human developmental disease that affects 1 of every 1000 newborns (58) and underscore the relevance of studying TRIM71-mediated mRNA repression mechanisms.

### TRIM71 and NMD components cooperate in the repression of specific target mRNAs

NMD was first known for its role in the degradation of premature stop codon (PTC)-containing transcripts, but it is now known that NMD functions extend beyond mere quality control and participate in homeostatic regulation of gene expression. PTC-containing transcripts are targeted to NMD via the Exon Junction Complex (EJC) model (40), but the EJC-independent manner in which functional transcripts lacking PTCs are targeted to NMD remains poorly understood and has been typically observed to occur in mRNAs containing long 3′UTRs (13,14). Of note, CDKN1A mRNA contains a 1500 bp-long 3′UTR. Our results show that TRIM71-mediated CDKN1A 3′UTR repression was impaired upon knockdown of several NMD factors (SMG1, UPF1 and SMG7) in HEK293T cells, and endogenous CDKN1A mRNA levels were upregulated under the same conditions, suggesting that TRIM71 relies on NMD to mediate CDKN1A mRNA degradation. We found such NMD factors coprecipitated with overexpressed TRIM71 in HEK293T and with endogenous TRIM71 in HepG2 cells. The ubiquitylation mutant TRIM71 (C12LC15A) was also able to bind UPF1 in HEK293T and mES cells, consistent with the fact that this construct was properly located to P-bodies and significantly repressed CDKN1A 3′UTR. In contrast, our ΔNHL6 mutant, which neither bound CDKN1A mRNA nor repressed its 3′UTR, was likewise unable to bind UPF1. Furthermore, we also demonstrated that the interaction between TRIM71 and UPF1/SMG1 is RNA-dependent, suggesting that such an interaction is mediated by common mRNA targets and is therefore established within messenger ribonucleoprotein (mRNP) complexes. Of note, in a previous study, the interaction of the EJC-component Y14 with UPF1 was also markedly reduced upon RNase treatment (59).

Although CDKN1A/p21 is a known p53 target, our results confirmed that the regulation of CDKN1A induced by the TRIM71/NMD axis did not result from p53-mediated transcriptional activation in HEK293T cells. Furthermore, knockdown of TRIM71 or UPF1 in HepG2 cells led to CDKN1A mRNA upregulation but did not alter CDKN1A pre-mRNA levels, excluding a p53-independent transcriptional contribution. Moreover, knockdown of TRIM71 or UPF1 in HepG2 cells led to CDKN1A 3′UTR reporter derepression and increased CDKN1A mRNA stability after transcriptional inhibition with Actinomycin D, confirming that the TRIM71/NMD-dependent regulation of CDKN1A occurs post-transcriptionally via 3′UTR recognition followed by mRNA degradation in HepG2 cells.

Importantly, the upregulation of CDKN1A observed upon NMD impairment occurred only in the presence of TRIM71. Similarly, other TRIM71 mRNA targets (all of which contain a 3′UTR longer than 750 bp) were bound and regulated by UPF1 in a TRIM71-dependent manner, whereas UPF1-mediated regulation of classical EJC-dependent NMD targets was TRIM71-independent. The presence of a long 3′UTR resembles a PTC-like situation as explained by the ‘faux 3′UTR model’ described in *S. cerevisiae*, which postulates that translation termination occurring far upstream of the 3′end is intrinsically abnormal because it prevents the interaction between the poly-A-binding protein PABP and the release factor eRF3 bound to the terminating ribosome (60). In short 3′UTRs, such an interaction would prevent NMD based on a competition between UPF1 and PABP for binding to eRF3 (61), whereas aberrant translation termination in transcripts with PTCs or long 3′UTRs would result in the recruitment of the surveillance complex SURF, in which UPF1 and SMG1 join the release factors eRF1 and eRF3 to bind the terminating ribosome (59). After SURF recruitment, the EJC mediates the activation of NMD in PTC-containing transcripts, and similarly, other factors present in the mRNP complex must exert a EJC-like role for NMD activation in targets with long 3′UTRs. Our work identifies TRIM71 as one of these factors, which operates in a target-specific and cell-specific manner. Nonetheless, how TRIM71 mechanistically promotes the recruitment and/or activation of the NMD machinery for the degradation of specific targets remains to be further investigated. Given the RNA-dependent nature of the interaction between TRIM71 and NMD components, the recruitment of the NMD machinery to the mRNA could involve allosteric-dependent interactions that are stabilized only if one or both binding partners are bound to the RNA. Such interactions would thus occur only within the mRNP complex, where they are functionally relevant, whereas random interactions of these proteins in the cytosol would be prevented. Allosteric-dependent interactions have been described for other RNA-binding proteins within mRNPs (62,63). Alternatively, TRIM71 could enable the recruitment and/or activation of the NMD machinery indirectly by antagonizing NMD inhibitory molecules within the mRNP. Collectively, the competition between NMD stimulators (e.g. EJC, TRIM71) and inhibitors (e.g. PABP) within a given mRNP complex may ultimately determine the fate of the mRNA, underscoring the need for further research for the identification of other NMD regulators. This could also explain why some TRIM71 targets are indeed not regulated by NMD, since counteractive NMD inhibitory signals are most likely target-specific.

Altogether, we have investigated the molecular basis of TRIM71-mediated repression of CDKN1A mRNA in detail, identifying several TRIM71 responsive elements within the CDKN1A 3′UTR and demonstrating direct binding to one of them. Furthermore, we have characterized the TRIM71 domains required for mRNA binding, 3′UTR repression and proper subcellular localization, and we have confirmed that CDKN1A mRNA repression is miRNA- and AGO-independent. Our work has identified SMG1, UPF1 and SMG7 as new factors participating in TRIM71-mediated mRNA degradation, uncovering a role for TRIM71 in non-canonical NMD. Thus, our work lays the foundation for further studies investigating the *in vivo* implications of CDKN1A regulation mediated by the TRIM71/NMD axis in developmental and oncogenic pathologies, as well as identifying new targets regulated by this novel RNA surveillance pathway.

## Supplementary Material

gkz1057_Supplemental_Files
