# Supplementary material for: The mRNA repressor TRIM71 cooperates with Nonsense-Mediated Decay factors to destabilize the mRNA of CDKN1A/p21
Source: Nucleic Acids Res. 2019 Nov 16;47(22):11861–79. doi: 10.1093/nar/gkz1057 (PMC7145526; doi:10.1093/nar/gkz1057)
Supplement: gkz1057_Supplemental_Files [file gkz1057_supplemental_files.zip › Suppl.Methods_191022.pdf]

Supplementary Methods:

Generation of Stable Cell Lines:

For the stable expression of GFP or GFP-TRIM71, HEK293 cells, susceptible to Neomycin/G418 antibiotic, were transfected by Calcium Phosphate with linearized plasmids (pN1-GFP or pN1-GFP-TRIM71) containing a Neomycin resistance gene. The expression of GFP was confirmed 48 hours post transfection under the fluorescence microscope. Around 50% of the cells were shown to be efficiently transfected (data not shown). The integration of DNA into the genome occurs randomly in approximately 1 of every 10^5 transfected cells. Stably transfected cells (GFP positive) were then selected by incubation with 600ug/µl G418-BC antibiotic for 8 days, changing the antibiotic-containing media every second day. After selection, 100% of the cells were GFP positive and considered to be stably transfected. Individual clones were grown and the expression of GFP or GFP-TRIM71 was checked by protein extraction followed by western blot analysis with anti-GFP and anti-TRIM71 antibodies (See Antibodies Table).

The generation of HEK293T AGO2 Knockout cells was achieved by transfection with CRISPR/CAS9 vectors targeting the exon 3 of AGO2, starting at 309bp within the mature mRNA sequence (Target Sequence: 5'-GGAAGCCCGTGTTTGACGGCAGG-3'). Single clones were grown and deep-sequenced with primers amplifying the target sequence to confirm the presence of an ORF-altering mutation. Positive clones were grown and then checked for AGO2 expression by protein extraction followed by western blot analysis with anti-AGO2 antibody (See Antibodies Table).

Structural modeling of the human TRIM71 NHL domain and its interaction with RNA:

The protein sequences from zebrafish trim71/lin-41 and human TRIM71 share an overall identity of 84% over the 390 amino acids covering the FLN and NHL domains, corresponding to a sequence similarity of 96%. As the crystal structure of the zebrafish FLN-NHL domains bound to a 13-mer stem-loop RNA sequence has been determined recently (Kumari et al., 2018), we were able to model the human TRIM71 NHL domain in complex with the 13-mer stem-loop RNA that we identified in CDKN1A 3'UTR. Structural modelling of human TRIM71 FLN-NHL protein domains, residues 479-868, was perform using Swiss-Model (Waterhouse et al., 2018), based on the trim71/lin-41 crystal structure 6FQ3 which was determined at 1.9 Å resolution (Kumari et al., 2018). The 13-mer stem-loop RNA element GUCUUGUGAAGGC identified here to interact with the human NHL domain has been modelled on the RNA of the 6FQ3 complex by single nucleotide exchange with the program Coot (Emsley et al., 2010).

Production of recombinant vaccinia virus expressing human Flag-NHL protein:

The sequence of the human Flag-NHL protein was amplified by PCR from a previously generated pRK5 vector containing the full length TRIM71 sequence, and sub-cloned into a derivative of the vaccinia virus expression vector pTKG (Romeo and Seed, 1991). The generation of recombinant virus was done as previously described (Kolanus et al., 1993). In short, CV-1 cells were grown to maximal confluence and infected with a vaccinia virus WR wild type stock in DMEM media without supplements for 2h at 37°C. The media was replaced by DMEM supplemented with 10% FBS and 1% Pen/Strep antibiotic solution

and cells were then transfected by the Calcium Phosphate method (Graham and van der Eb, 1973) with the pTKG-Flag-NHL vector. Progression of infection was monitored by visual inspection and fully rounded cells were harvested and lysed in 10mM Tris pH 9 for 30min at RT to recover recombinant viruses. Purification of recombinant viruses was done by several infection-selection rounds in selective DMEM media (250 µg/ml Xanthine, 15 µg/ml hypoxanthine, 25 µg/ml mycophenolic acid and 2,5% FCS). Surviving viruses were isolated 24-48h post infection from single plaques, and used for a new infection. The purification of the recombinant Flag-NHL virus ended after 4-5 selection rounds, when all plaques within a plate were stained positive with a AP-conjugated anti-Flag antibody.

Production of recombinant human Flag-NHL protein:

30x10cm dishes of confluent CV-1 cells were infected with recombinant Flag-NHL virus and 48h post-infection, cells were harvested and lysed in 5ml of RIPA buffer for 30min on ice. The lysate (around 100mg of total protein) was then cleared by centrifugation and incubated with 200µl of M2 anti-FLAG magnetic beads rotating at 4°C overnight for the immunoprecipitation of Flag-NHL protein. After their overnight incubation, beads were washed 5 times for 10 min each with 2ml TBS buffer. Flag-NHL protein was then eluted from the beads in its native conformation by adding an excess of 3xFLAG peptide. Two elution steps were performed subsequently with 500 µg/ml 3XFLAG peptide in 500 µl TBS. Each elution step was conducted for 30min by incubation in rotation at 4°C. The two eluates were then mixed, and centrifuged for 30min in a 10KDa size exclusion column to get rid of the 3XFLAG peptide and concentrate the produced protein. The protein purity and integrity were checked on a 12% PAGE-SDS gel followed by Coomassie Blue staining (See Fig. 3D), and protein concentration was estimated from a Coomassie staining-standard curve.

References:

Emsley *et al.,* (2010). Features and development of Coot. *Acta Crystallogr D Biol Crystallogr.* 66 (Pt 4):486-501.

Graham & Van Der Eb (1973). A new technique for the assay of infectivity of human adenovirus 5 DNA. *Virology*, 52;456.

Kolanus *et al.,* (1993). T cell activation by clustered tyrosine kinases. *Cell* 16;74(1):171-83.

Kumari *et al.,* (2018). Evolutionary plasticity of the NHL domain underlies distinct solutions to RNA recognition. *Nat Commun*. 19;9(1):1549.

Romeo & Seed, (1991). Cellular immunity to HIV activated by CD4 fused to T cell or Fc receptor polypeptides. *Cell*. 8;64(5):1037-46.

Waterhouse *et al.,* (2018) SWISS-MODEL: homology modelling of protein structures and complexes. *Nucleic Acids Res*. 2;46(W1):W296-W303.

Supplementary Material Tables

Cell Reagents

Name	Company	Product ID
RPMI 1640	PAN Biotech	P04-17500
DMEM	PAN Biotech	P04-01549
PBS	PAN Biotech	P04-35500
Penicillin-Streptomycin	PAN Biotech	P06-07100
G418-BC	Biochrom	A 2912
DMEM knockout	Gibco	10389172
Fetal Bovine Serum (FBS)	Sigma-Aldrich	F2442
Non-essential aminoacids (NEAA)	Sigma-Aldrich	M7145
β-mercaptoethanol	Sigma-Aldrich	M6250
GlutaMAX	Thermo Fisher Scientific	35050061
Lipofectamine 2000	Thermo Fisher Scientific	11668027
Lipofectamine RNAiMAX	Thermo Fisher Scientific	13778075
PANfect	PAN Biotech	P02-8010
Cell Proliferation Dye eFluor 670	Thermo Fisher Scientific	65-0840-85
Actinomycin D	Sigma-Aldrich	A9415-2MG
DMSO	Roth	A994

Commercial Kits

Name	Company	Product ID
Pierce BCA Protein Assay Kit	Thermo Fisher Scientific	23225
Pierce ECL Western Blotting Substrate	Thermo Fisher Scientific	32106
cDNA Reverse Transcription Kit	Thermo Fisher Scientific	4368814
peqGOLD TriFast	PeqLab	30-2010
Dual-Luciferase Reporter Assay System	Promega	E1910
iTaq Universal SYBR Green Supermix	BIO-RAD	172-5122
iTaq Universal Probes Supermix	BIO-RAD	172-5132

Antibodies

Name	Company	Product ID
Rabbit anti-TRIM71 (93,4 kDa)	Sigma-Aldrich	HPA038142
Sheep anti-LIN-41 (93,4 kDa)	R & D Systems	AF5104
Rabbit anti- p21 (12D1) (18,1 kDa)	Cell Signaling Technology	2947
Rabbit anti-DGCR8 (86 kDa)	Proteintech Group	10996-1-AP
Rat-anti-AGO2 (93,6 kDa)	Sigma-Aldrich	SAB4200085
Rabbit anti-ZCCHC11 (TUT4) (185 kDa)	Proteintech Group	18980-1-AP
Rabbit anti-UPF1 (123 kDa)	Cell Signaling Technology	9435S
Rabbit anti-SMG1 (410 kDa)	Cell Signaling Technology	4993
Rabbit anti-SMG7 (127 kDa)	Bethyl	A302-170A-M
Rabbit anti-SMG7 (for IP)	Bethyl	A302-170A
Rabbit anti-P53 (43,6 kDa)	Cell Signaling Technology	2527
Mouse anti-p-P53 (Ser15) (43,7 kDa)	Cell Signaling Technology	9286
Rabbit anti-p-H2AX (Ser139) (15,8 kDa)	Cell Signaling Technology	2577
Rabbit anti-β-ACTIN (41,7 kDa)	Sigma-Aldrich	A2066
Mouse anti-α-TUBULIN (50,1 kDa)	Sigma-Aldrich	T9026
Mouse anti-VINCULIN (123,8 kDa)	Sigma-Aldrich	V9131
Mouse anti-GAPDH (36 kDa)	Acris	ACP001P
Goat anti-IgG (39,7 kDa)	Jackson ImmunoResearch	109-005-098
Mouse anti-GFP (32,7 kDa)	Santa Cruz Biotechnology	sc-9996
Dynabeads Protein G	Thermo Fisher Scientific	10004D
Anti-FLAG M2 Magnetic Beads	Sigma-Aldrich	M8823
Mouse anti-FLAG M2	Sigma-Aldrich	F1804

Cloning Primers

Name	Sequence (5'-3')
Hs_Trim32_For_MluI	GCGGGGACGCGTATGGCTGCAGCAGCAGCTTC
Hs_Trim32_Rev_NotI	GCGGGGGCGGCCGCCTATGGGGTGGAATATCTTCTCAGATGG
Hs_Trim71_For_MluI	GGGGCGACGCGTATGGCTTCGTTCCCCGAGACC
Hs_Trim71_Rev_NotI	GGGGCGGCGGCCGCTTTAGAAGACGAGGATTCGATTGTTGCC
Hs_Trim71_ΔNHL6_Rev_NotI	GGGGCGGCGGCCGCTTAGTTGGATTCAAACATCTGTACCCGA
Hs_Trim71_RBB_Rev_NotI	GCGGGGGCGGCCGCTTAGCGGATCTTTTCTACCTTCCACAG
Hs_Trim71_CCNHL_For_MluI	GGGGCGACGCGTCAGGAGGCACTGCAGGACTC
Hs_Trim71_FLNNHL_For_MluI	GGGGCGACGCGTTTTGTTAGCAGCGGGGCCT
Hs_Trim71_NHL_For_MluI	GGGGCGACGCGTAAGTCAGGCCGCAGC
Hs_Trim71_C12LC15A_For_QCM	CCGATTTCCAGATCTTATTGCTGGCAAAGGAGATGTGCGGC
Hs_Trim71_C12LC15A_Rev_QCM	GCCGCACATCTCCTTTGCCAGCAATAAGATCTGGAAATCGG
Hs_Trim71_ΔCC_For (FBS)_SOEing	GTAGAAAAGATCCGCCTAGACATCCTACTGGCCCG
Hs_Trim71_ΔCC_Rev (BB2)_SOEing	CAGTAGGATGTCTAGGCGGATCTTTTCTACCTTCCAC
Hs_DCP1A_For_MluI	GGGGCGACGCGTATGGAGGCGCTGAGTCGAG
Hs_DCP1A_Rev_NotI	GGGGCGGCGGCCGCTCATAGGTTGTGGTTGTCTTTGTTCTTGG
Hs_UPF1_For_MluI	GGGGCGACGCGTATGAGCGTGGAGGCGTACG
Hs_UPF1_Rev_NotI	GGGGCGGCGGCCGCTTAATACTGGGACAGCCCCGTC
Hs_CDKN1A_3'UTR_For_FL/F1_XhoI	GGGGCGCTCGAGTCCGCCCACAGGAAGCCTGCAGTC
Hs_CDKN1A_3'UTR_Rev_FL/F3_NotI	GGGGCGCGGCCGCACAAGTAAAGTCACTAAGAATCATTTATTGAG
Hs_CDKN1A_3'UTR_Rev_F1_NotI	GGGGCGGCGGCCGCTTTGATGATGCCCCCACTCGG
Hs_CDKN1A_3'UTR_For_F2_XhoI	GGGGCGCTCGAGAACTTTGGAGTCCCCTCACCTC
Hs_CDKN1A_3'UTR_Rev_F2_NotI	GGGGCGGCGGCCGCCCTGTGCCACCACATGGGA
Hs_CDKN1A_3'UTR_For_F3_XhoI	GGGGCGCTCGAGCCCCCTTGAGTGGGGTTATCT
Hs_CDKN1A_3'UTR_Rev_F2_0-400_NotI	GGGGCGGCGGCCGCCCTTCCAGTCCATTGAGCTGG
Hs_CDKN1A_3'UTR_Rev_F2_0-300_NotI	GGGGCGGCGGCCGCGGAATTGCAGAGCCCAGCTG
Hs_CDKN1A_3'UTR_Rev_F2_0-200_NotI	GGGGCGGCGGCCGCCTCAACACTGAGACGGGCTCC
Hs_CDKN1A_3'UTR_Rev_F2_0-100_NotI	GGGGCGGCGGCCGCGTATCAAGAGCCAGGAGGGTACC
Hs_CDKN1A_3'UTR_For_F2_100-200_XhoI	GGGGCGCTCGAGGGTACCCTCCTGGCTCTTGATAC
psiCHECK2_NewNotISite_For_QCM	CAGTAATTCTAGGCGGCCGCTCGAGCGCCAAC
psiCHECK2_NewNotISite_Rev_QCM	GTTGGCGCTCGAGCGGCCGCCTAGAATTACTG
psiCHECK2_F2_100-200_M#1_For_QCM	GCTCTTGATACCCCCCTCTAGGGGGAAGGTGGGG
psiCHECK2_F2_100-200_M#1_Rev_QCM	CCCCACCTTCCCCCTAGAGGGGGGTATCAAGAGC
psiCHECK2_F2_100-200_M#2_For_QCM	CCCCCTCTGTCTTGTAAAGGCAGGGGGAAGG
psiCHECK2_F2_100-200_M#2_Rev_QCM	CCTTCCCCCTGCCTTTACAAGACAGAGGGGG
psiCHECK2_F2_100-200_M#3_For_QCM	CCCCCTCTGTCTTGTCAAGGCAGGGGGAAGG
psiCHECK2_F2_100-200_M#3_Rev_QCM	CCTTCCCCCTGCCTTGACAAGACAGAGGGGG
psiCHECK2_F2_100-200_M#4_For_QCM	CCCCCTCTGTCTTGTGCAGGCAGGGGGAAGG
psiCHECK2_F2_100-200_M#4_Rev_QCM	CCTTCCCCCTGCCTGCACAAGACAGAGGGGG

Primers for SYBR-Green qPCR

Name	Sequence (5'-3')
Hs_18S rRNA Fw	GTAACCCGTTGAACCCCATTC
Hs_18S rRNA Rev	CCATCCAATCGGTAGTAGCGAC
Hs_PUM2 Fw	ATGTCCCAGCCTATTATGGTACAG
Hs_PUM2 Rev	CCTTTCTCAGGTCCATCTGTTTCAG
Hs_SMG1 Fw	TACTTATGGTCGGAAGTCGTTGG
Hs_SMG1 Rev	GCCAATCTTCGGTCTCTGTCG
Hs_SMG6 Fw	CGGGAGCAGAGAAAACATGAA
Hs_SMG6 Rev	AGCAGAGCAATCTCGGTCAT
Hs_SMG7 Fw	TACCTCCGGCAGGCAGAA
Hs_SMG7 Rev	CTGGCCTTGCAGTGTTGTGA

Primers for SYBR-Green qPCR

Name	Sequence (5'-3')
Hs_ATF3 Fw	CACTGGTGTTTGAGGATTTTGCTAA
Hs_ATF3 Rev	GCAGCTGCAATCTTATTTCTTTCTC
Hs_TBL2 Fw	CATCTGGAGCACCAAGGACTTC
Hs_TBL2 Rev	GTGCTTTTTAGGGAAGTCCTCTGG
Hs_GADD45B Fw	GCAGAAGATGCAGACGGTGAC
Hs_GADD45B Rev	CACGATGTTGATGTCGTTGTCAC
Hs_pre-CDKN1A Fw	ACTCTCAGGGTCGAAAACGG
Hs_pre-CDKN1A Rev	GCCTGGCATAATGAACATTCCC
Hs_HOXA5 Fw	AAAACTCCCTAAGCAACTCC
Hs_HOXA5 Rev	ATGTTGTCATGACTTATGTGC
Hs_MYB Fw	GGAGACCCCGACACAGC
Hs_MYB Rev	CCAAGTGACGCTTTCCAGATTTG
Hs_STATB5 Fw	CCCAGCGCAGGCAACT
Hs_STATB5 Rev	AGCGGTCATACGTGTTCTGG
Hs_CCNE2 Fw	TAGCTGGTCTGGCGAGGTTT
Hs_CCNE2 Rev	ACAGGTGGCCAACAATTCCT
Hs_CDH1 Fw	GAAAACAGCAAAGGGCTTGGA
Hs_CDH1 Rev	TTAGGGCTGTGTACGTGCTG

Probes for Taqman qPCR All from Thermo Fisher Scientific

Name	Product ID
Hs_CDKN1A (TaqMan® Gene Expression Assays)	Hs00355782_m1
Hs_TRIM71 (TaqMan® Gene Expression Assays)	Hs01394933_m1
Hs_UPF1 (TaqMan® Gene Expression Assays)	Hs00161289_m1
Hs_HMGA2 (TaqMan® Gene Expression Assays)	Hs00171569_m1
Hs_HPRT1 (TaqMan® Gene Expression Assays)	Hs01003267_m1
Hs_FOXJ1 (TaqMan® Gene Expression Assays)	Hs00230964_m1
Hs_E2F7 (TaqMan® Gene Expression Assays)	Hs00171569_m1
Hs_INHHB (TaqMan® Gene Expression Assays)	Hs00173582_m1
Hs_GADD45A (TaqMan® Gene Expression Assays)	Hs01003267_m1
hsa-let-7a (TaqMan® miRNA Assays)	000377
mmu-miR-294 (TaqMan® miRNA Assays)	001056
U6 snRNA (TaqMan® miRNA Assays)	001973

siRNAs

Name	Sequence (5'-3')
siCtrl	AAACAUGCAGAAAAUGCUGTT
siTRIM71#1	CCGUGUGCGACCAGAAAGUATT
siTRIM71#2	AGAAAGUAGUGCUAGCCGATT
siDGCR8	AUCCGUUGAUCUCGAGGAAUUTT
siAGO2	GCACGGAAGUCCAUCUGAAUUTT
siPUM2	GCAUGGUAGAAUAUGUAUUTT
siTUT4 (ZCCHC11)	GGAGCACAUAAACAUAUAATT
siUPF1#1	GAUGCAGUUCCGCUCCAUUTT
siUPF1#2	AAUUUCUGUAACUUGUUUCCUTT
siSMG1	GUGAAGAUGUUCCCUAUGATT
siSMG6	GGGUCACAGUGCUGAAGUATT
siSMG7	CAGCACAGUCUACAAGCCATT

**5’Cy3-NHS-ssRNA**	All from Elle Biotech
**Name**	**Sequence (5'-3')**
SL WT	GCUUUGUGAAGGC
SL M#3	GUCUUGUCAAGGC

**mirVana miRNA Mimics**	All from Thermo Fisher Scientific
**Name**	**Product ID**
Negative Control #1 (miR Ctrl)	4464058
Let-7a	4464066

Enzymes & Inhibitors

Name	Company	Product ID
peqGOLD Proteinase K	Peqlab	04-1071
DNaseI	Thermo Fisher Scientific	EN0521
RNase A	Thermo Fisher Scientific	EN0531
RiboLock RNase Inhibitor	Thermo Fisher Scientific	EO0381
SIGMAFAST™ Protease Inhibitor Tablets	Sigma-Aldrich	S8820

Softwares

Name	Company	Product ID
CFX Manager Software for qPCR Analysis	BIO-RAD	1845000
FACS Diva Software for FACS Data Recording	BD Biosciences	659523
FlowJo V10 Software for FACS data Analysis	FlowJo	RRID:SCR_008520
Olympus Fluoview FV10-ASW Software	Olympus	RRID:SCR_014215
Fiji (Image J) for Microscopy data analysis	Fiji	RRID:SCR_002285
WinGlow for Luminiscence measurement	Berthold Technologies	
Prism7 for data illustration and analysis	GraphPad	
