# Supplementary material for: The mRNA repressor TRIM71 cooperates with Nonsense-Mediated Decay factors to destabilize the mRNA of CDKN1A/p21
Source: Nucleic Acids Res. 2019 Nov 16;47(22):11861–79. doi: 10.1093/nar/gkz1057 (PMC7145526; doi:10.1093/nar/gkz1057)
Supplement: gkz1057_Supplemental_Files [file gkz1057_supplemental_files.zip › Supplementary Figures_191022.pdf]

Figure S1

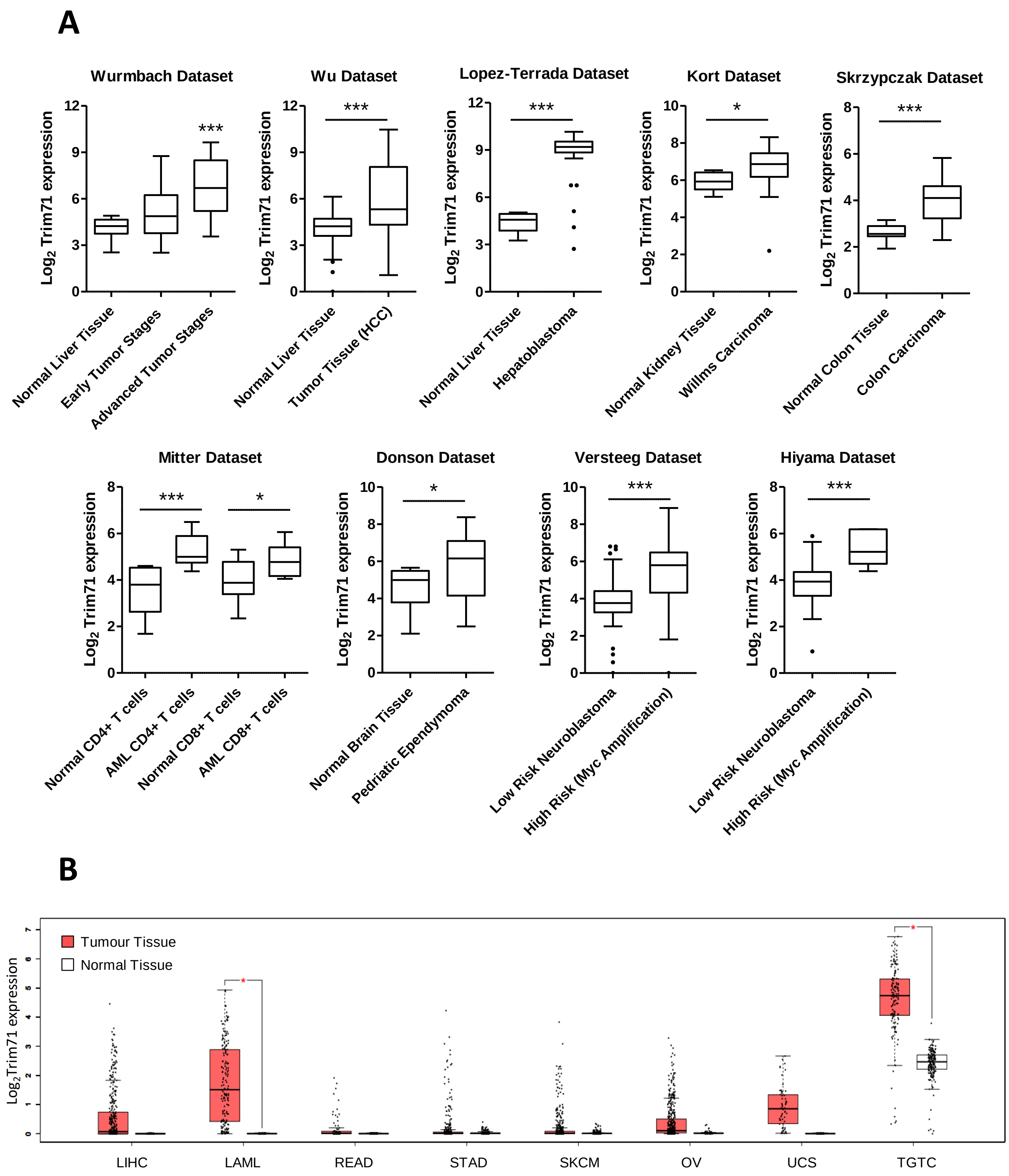

A
Wurmbach Dataset
Wu Dataset
Lopez-Terrada Dataset
Kort Dataset
Skrzypczak Dataset
Log2 Trim71 expression
Normal Liver Tissue
Early Tumor Stages
Advanced Tumor Stages
Tumor Tissue (HCC)
Hepatoblastoma
Normal Kidney Tissue
Willms Carcinoma
Normal Colon Tissue
Colon Carcinoma
Mitter Dataset
Donson Dataset
Versteeg Dataset
Hiyama Dataset
Normal CD4+ T cells
AML CD4+ T cells
Normal CD8+ T cells
AML CD8+ T cells
Normal Brain Tissue
Pediatric Ependymoma
Low Risk Neuroblastoma
High Risk (Myc Amplification)
B
Tumour Tissue
Normal Tissue
Log2Trim71 expression
LIHC
LAML
READ
STAD
SKCM
OV
UCS
TGTC

Supplementary Figure 1: A) TRIM71 mRNA upregulation in several cancer types according to data from the R2 Genomics Analysis and Visualization Platform (http://r2.amc.nl/). The name/ID of the dataset employed to produce each graph is indicated on top of the respective graph. Graphs are depicted as Tukey Box-Plots. Statistical significance was calculated with a two-tailed unpaired t test: *pvalue<0.05; ***p value<0.005. **B)** TRIM71 mRNA upregulation in several cancer types according the GEPIA Server (http://gepia.cancer-pku.cn/). LIHC = Liver hepatocellular carcinoma. LAML = Acute Myeloid Leukemia. READ = Rectum adenocarcinoma. STAD = Stomach adenocarcinoma. SKCM = Skin Cutaneous Melanoma. OV = Ovarian serous cystadenocarcinoma. UCS = Uterine Carcinosarcoma. TGCT = Testicular Germ Cell Tumors. Graphs and statistics are depicted as provided by the server.

Figure S2

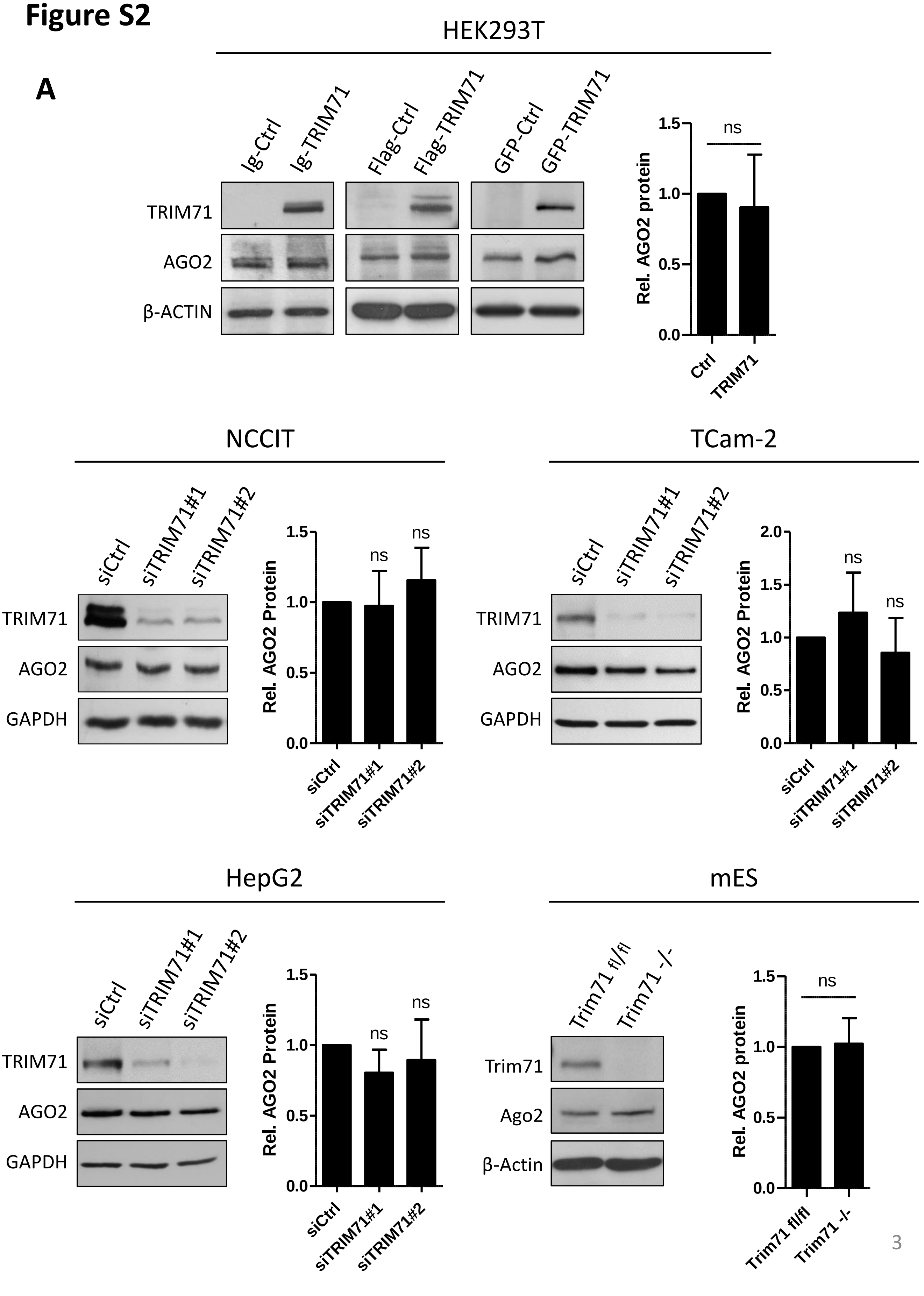
A
HEK293T
Ig-Ctrl
Ig-TRIM71
Flag-Ctrl
Flag-TRIM71
GFP-Ctrl
GFP-TRIM71
TRIM71
AGO2
β-ACTIN
Rel. AGO2 protein
ns
Ctrl
TRIM71
NCCIT
siCtrl
siTRIM71#1
siTRIM71#2
TRIM71
AGO2
GAPDH
Rel. AGO2 Protein
ns
TCam-2
HepG2
mES
Trim71 fl/fl
Trim71 -/-
Trim71
Ago2
β-Actin

B

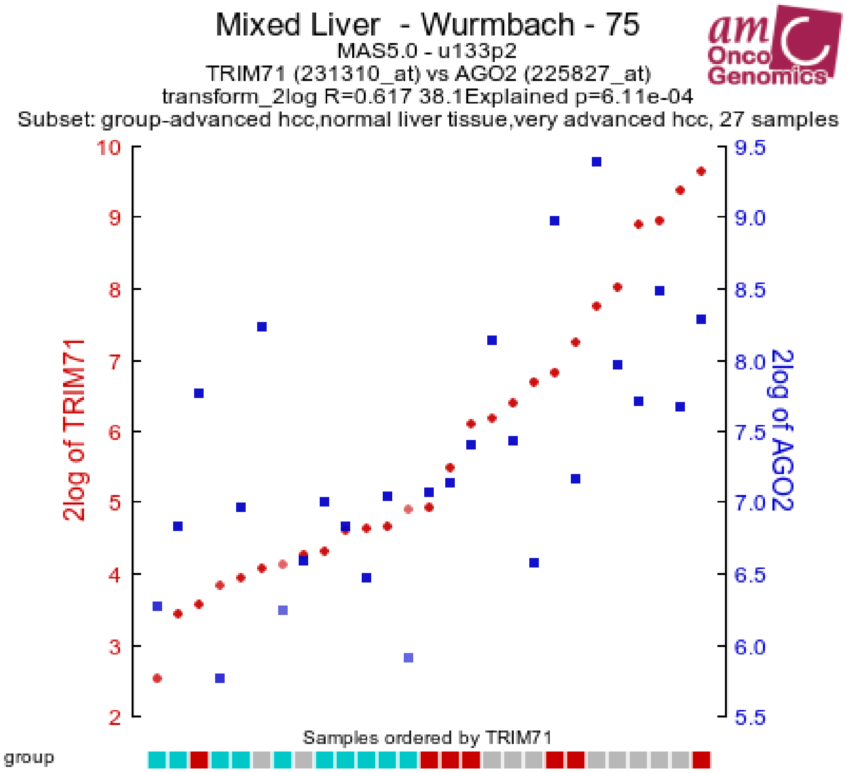

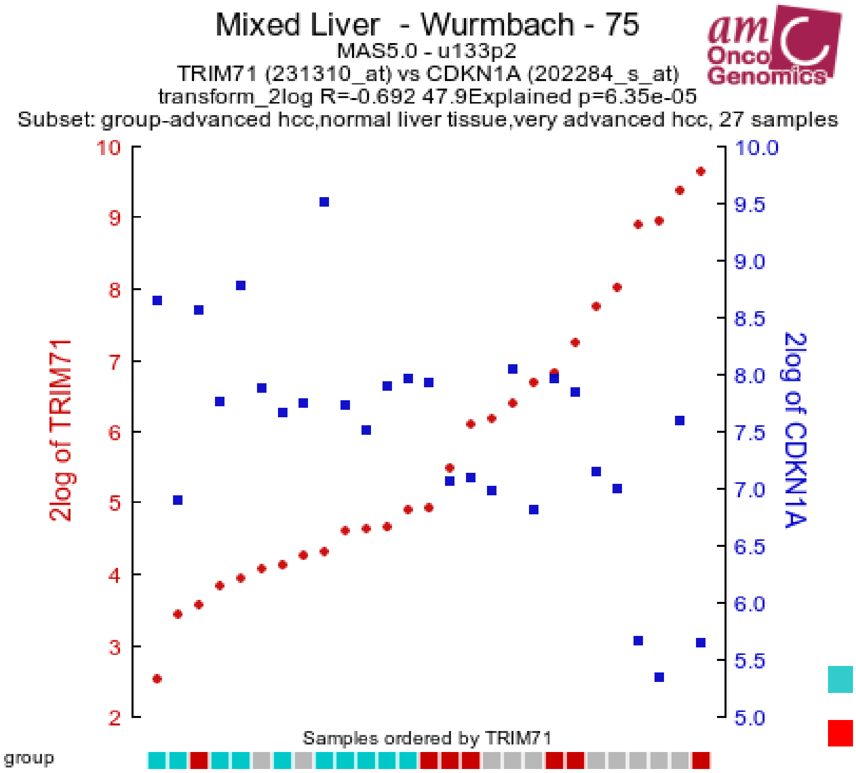

Normal Liver Tissue

Advanced HCC

Very Advanced HCC

Supplementary Figure 2: A) Representative immunoblots and AGO2 bands densitometry quantification showing unchanged AGO2 protein levels upon TRIM71 overexpression in HEK293T cells, TRIM71 knockdown in embryonic carcinoma (EC) cell lines NCCIT (non-seminoma subtype model) and TCam-2 (seminoma subtype model), TRIM71 knockdown in the hepatocellular carcinoma (HCC) cell line HepG2 and TRIM71 knockout in mES cells, as indicated on top of each figure. Graphs represent Mean±SD (for each cell line n=3, except for HEK293T in which n=8). **B)** Positive correlation between TRIM71 and AGO2 mRNA expression (top) and negative correlation between TRIM71 and CDKN1A mRNA expression (bottom) in samples from advanced stage-HCC patients (Source: R2 Genomics Analysis and Visualization Platform (http://r2.amc.nl), Wurmbach Dataset).

Figure S3 (Related to Fig. 1)

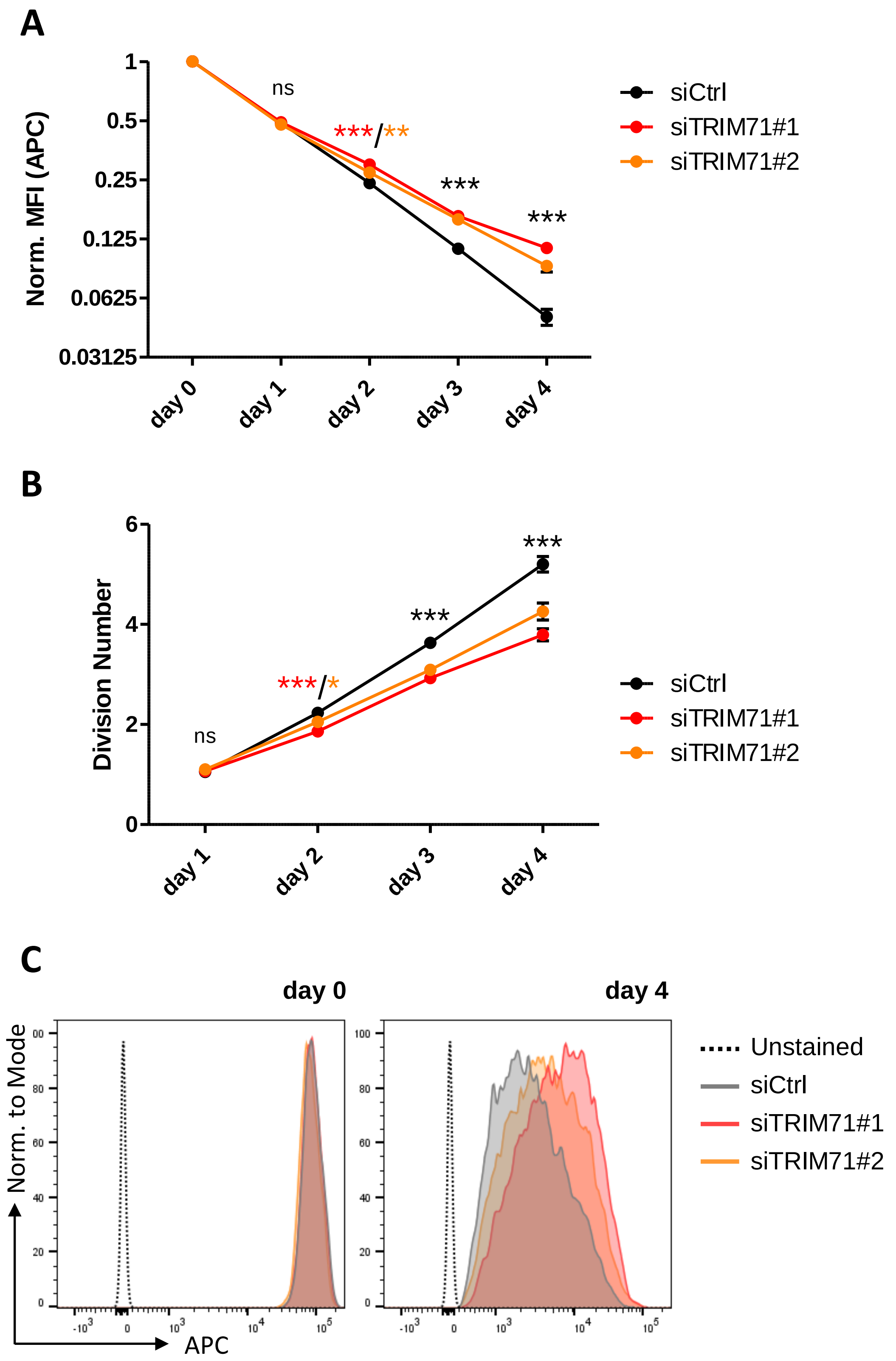

Supplementary Figure 3 (Related to Fig. 1): A) Progressive loss of fluorescence intensity over time of the dye eFluor670 upon proliferation of control (siCtrl) and TRIM71 knockdown (siTRIM71#1 and #2) HepG2 cells. MFI = Median Fluorescence Intensity of APC (eFluor670). **B)** Number of cell divisions undergone overtime by control and TRIM71 knockdown HepG2 cells. The number of divisions was calculated assuming that the MFI decreases by half upon each cell division with the following formula: Division Number = $\log_2 [(MFI_{day0} - MFI_{unstained})/(MFI_{dayX} - MFI_{unstained})]$. The average cell cycle duration was calculated from the number of cell divisions at day 4 and plotted as an individual graph (See Figure 1D). **C)** Overlap of APC (eFluor670) histograms of control and TRIM71 knockdown HepG2 cell populations showing comparable staining for the different populations at the beginning of the proliferation assay (left panel: day 0) and the retardation of TRIM71 knockdown histograms as result of a diminished proliferation at the end of the experiment (right panel: day 4). A-C show the results of one representative experiment out of four, each of them including three technical replicates. Graphs represent Mean±SD. Statistical significance was calculated with a two-tailed unpaired t test: *pvalue<0.05; **pvalue<0.01; ***pvalue<0.005.

Figure S4 (Related to Fig. 1)

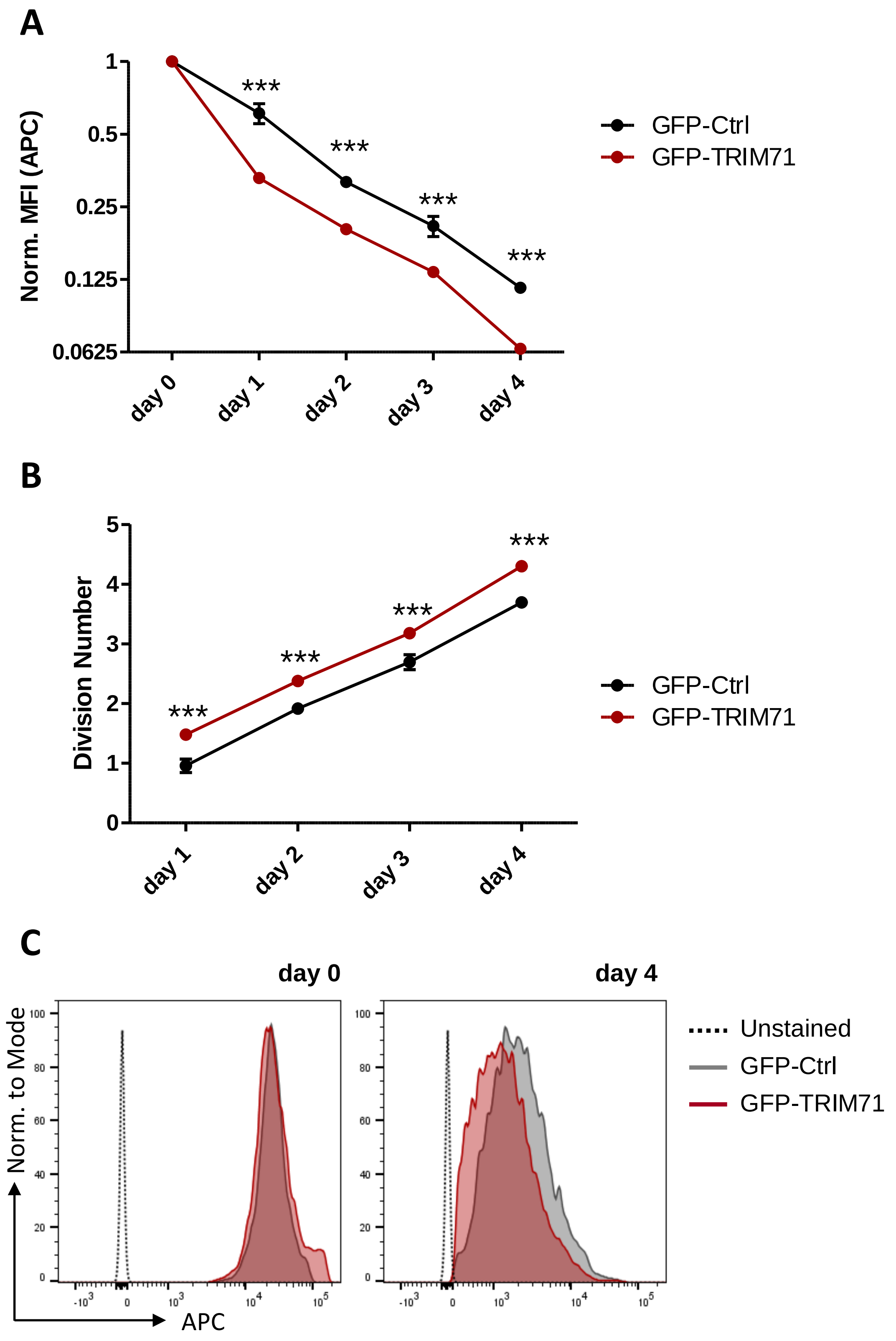

Supplementary Figure 4 (Related to Fig. 1): A) Progressive loss of fluorescence intensity over time of the dye eFluor670 upon proliferation in control (GFP-Ctrl) and TRIM71 (GFP-TRIM71) overexpressing HEK293 cells. MFI = Median Fluorescence Intensity of APC (eFluor670). **B)** Number of cell divisions undergone overtime by control and TRIM71 overexpressing HEK293 cells. The number of divisions was calculated as specified in Fig. S3B. The average cell cycle duration was calculated from the number of cell divisions at day 4 and plotted as an individual graph (See Fig. 1H). **C)** Overlap of APC (eFluor670) histograms of control and TRIM71 overexpressing HEK293 cell populations, showing comparable staining for the different populations at the beginning of the proliferation assay (left panel: day 0), and the advanced GFP-TRIM71 histogram as result of an enhanced proliferation at the end of the experiment (right panel: day 4). A-C show the results of one representative experiment out of three, each of them including three technical replicates. Graphs represent Mean±SD. Statistical significance was calculated with a two-tailed unpaired t test: *pvalue<0.05; **pvalue<0.01; ***pvalue<0.005.

Figure S5 (Related to Fig. 3)

A

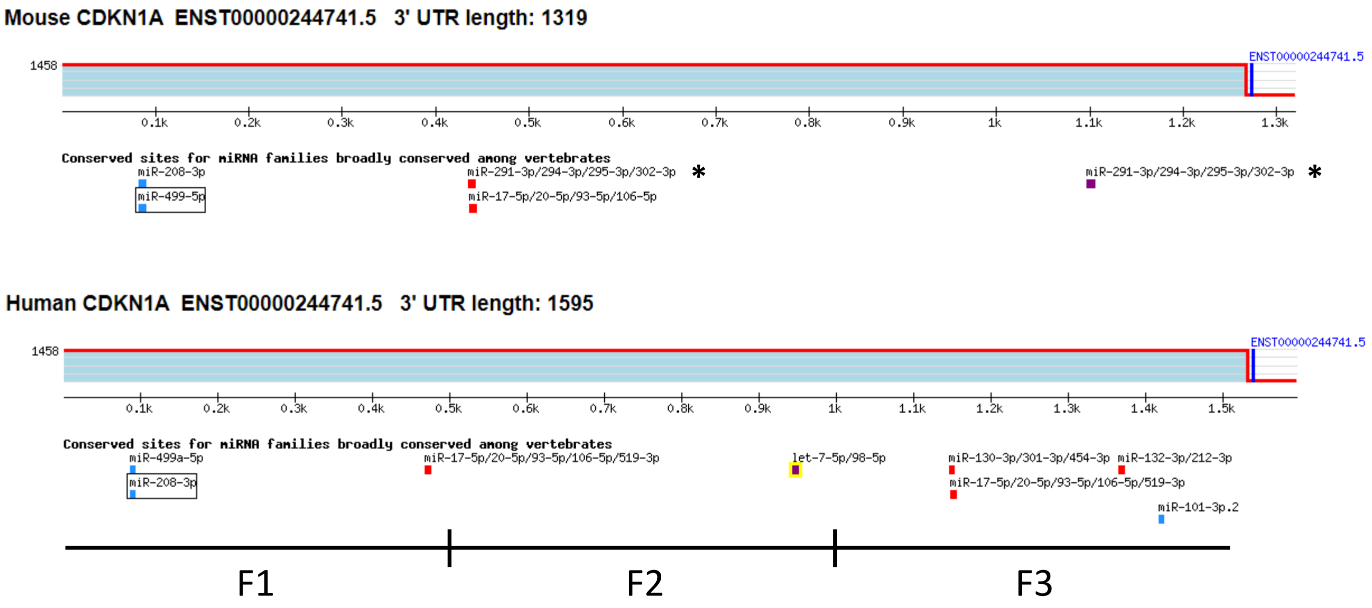

B

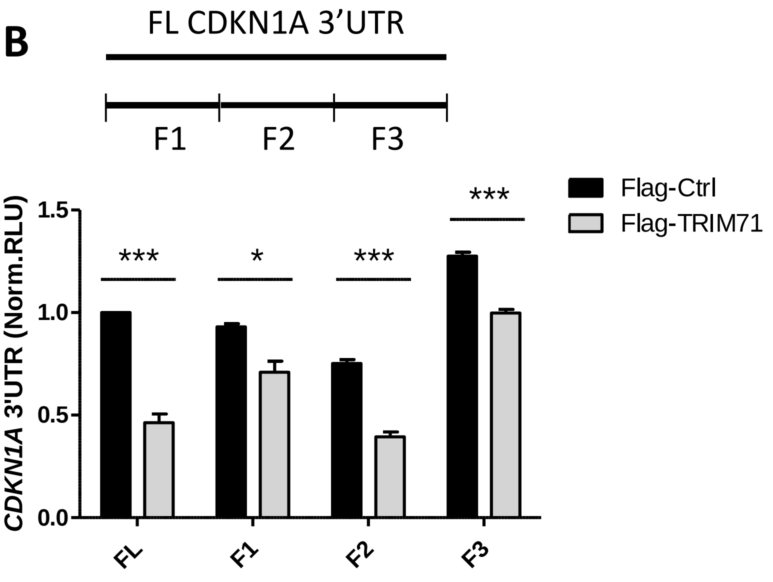

C

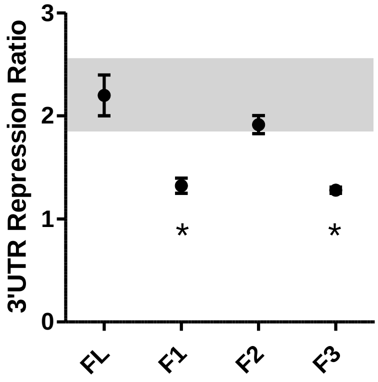

D

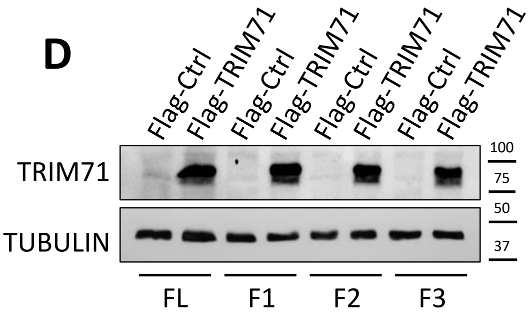

E

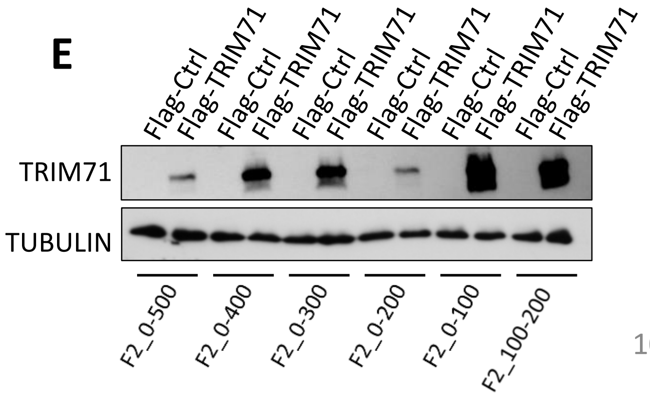

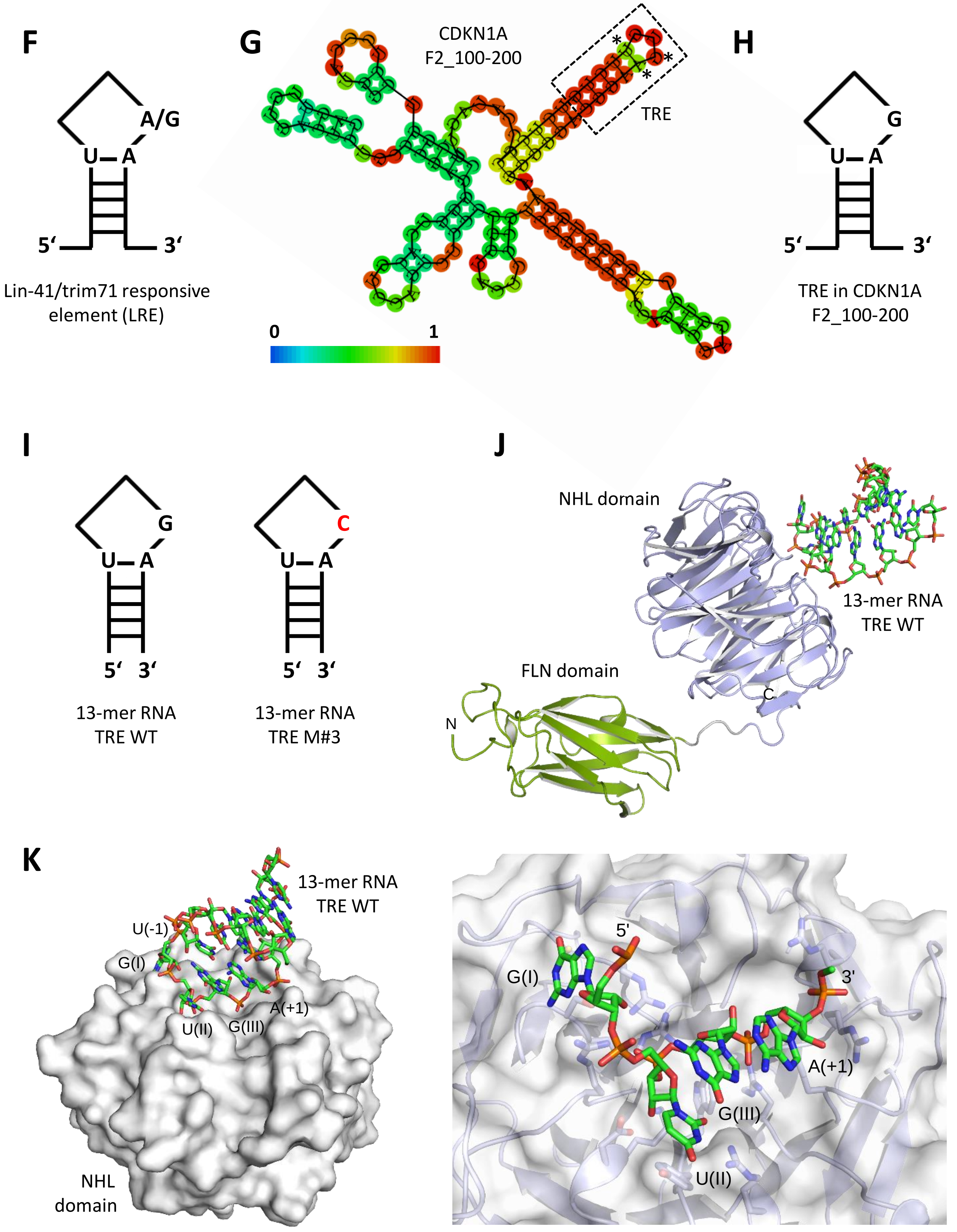
F
A/G
U A
5' 3'
Lin-41/trim71 responsive element (LRE)
G
CDKN1A F2_100-200
TRE
0
1
H
G
U A
5' 3'
TRE in CDKN1A F2_100-200
I
G
U A
5' 3'
13-mer RNA TRE WT
C
U A
5' 3'
13-mer RNA TRE M#3
J
NHL domain
13-mer RNA TRE WT
FLN domain
N
C
K
13-mer RNA TRE WT
U(-1)
G(I)
U(II)
G(III)
A(+1)
NHL domain
5'
3'
G(I)
A(+1)
G(III)
U(II)

Supplementary Figure 5 (Related to Fig. 3) A) TargetScan prediction of conserved miRNA binding sites (http://www.targetscan.org/) in mouse (up) and human (down) CDKN1A 3'UTR. Of note, two miRNA binding sites for the ES-specific miRNA family miR290-302 (*****) are present in the mouse 3'UTR but absent in the human 3'UTR. **B)** Repression of a luciferase reporter under the control of different CDKN1A 3'UTR fragments (depicted above the graph) in HEK293T cells transiently transfected with Flag or Flag-TRIM71. FL=full length CDKN1A 3'UTR (1536bp). F1=Fragment 1 (507bp), spanning from 1-507bp within the FL 3'UTR. F2=Fragment 2 (510bp), spanning from 508-1017bp within the FL 3'UTR. F3=Fragment 3 (519bp), spanning from 1018-1536bp within the FL 3'UTR. Graph represents Mean±SD (n=3). **C)** Repression ratio representing the capability of the Flag-TRIM71 full length wild type construct to repress the different fragments (F1-3) of the CDKN1A 3'UTR, calculated from data depicted in B, as follows: Repression $\text{Ratio}_{\text{Flag-ConstructX}} = \text{norm.RLU}_{\text{Flag-empty}} / \text{norm.RLU}_{\text{Flag-Construct X}}$. Graph represent Mean±SEM while the grey-shaded area represents the SD of the TRIM71-mediated repression of the control (FL) reporter. For A and B, statistical significance was calculated with a two-tailed unpaired t test: *pvalue<0.05; ***pvalue<0.005. **D)** Representative immunoblot showing TRIM71 expression for the luciferase assays depicted in B-C. **E)** Representative immunoblot showing TRIM71 expression for luciferase assays depicted in main Fig. 3A-B. **F)** Schematic representation of Lin-41/trim71 responsive element (LRE) identified by Kumari *et. al.,* 2018, consisting of a 13-mer stem-3base-loop RNA structure with specific nucleotides in conserved positions, namely A/G in loop position III, U in stem position -1 respective to the loop and A in stem position +1 respective to the loop, according to previous nomenclature. **G)** *In silico* prediction of the secondary structure for the CDKN1A 3'UTR F2_100-200 RNA sequence with RNAfold tool (http://rna.tbi.univie.ac.at/cgi-bin/RNAWebSuite/RNAfold.cgi), showing a LRE-like motif to be present in this human TRIM71 target, and from now called TRIM71 responsive element (TRE). TRE is enclosed by the dash-lined-box in which the nucleotides in positions III, -1 and +1 have been marked (*). The color bar represents nucleotide position probability. **H)** Schematic representation of TRE identified in G. **I)** Schematic representation of TRE ssRNAs used for fluorescent polarization experiments depicted in main Fig. 3E-F. **J)** Structural model of the Filamin (FLN) and NHL domains for human TRIM71 (479-868) based on the *D. Rerio* trim71/lin41 crystal structure of those domains (84% sequence identity). The model is shown in cartoon representation. **K)** Model of the human NHL domain in complex with the TRE WT RNA represented in I. The TRIM71 protein is shown as surface display and the RNA in stick representation. A positively charged binding pocket accommodates the phosphate backbone groups of the loop region, being direct contacts of the protein mediated to the three nucleotides of the loop region G(I), U (II) and G(III), and the succeeding A(+1).

Figure S6 (Related to Fig. 4)

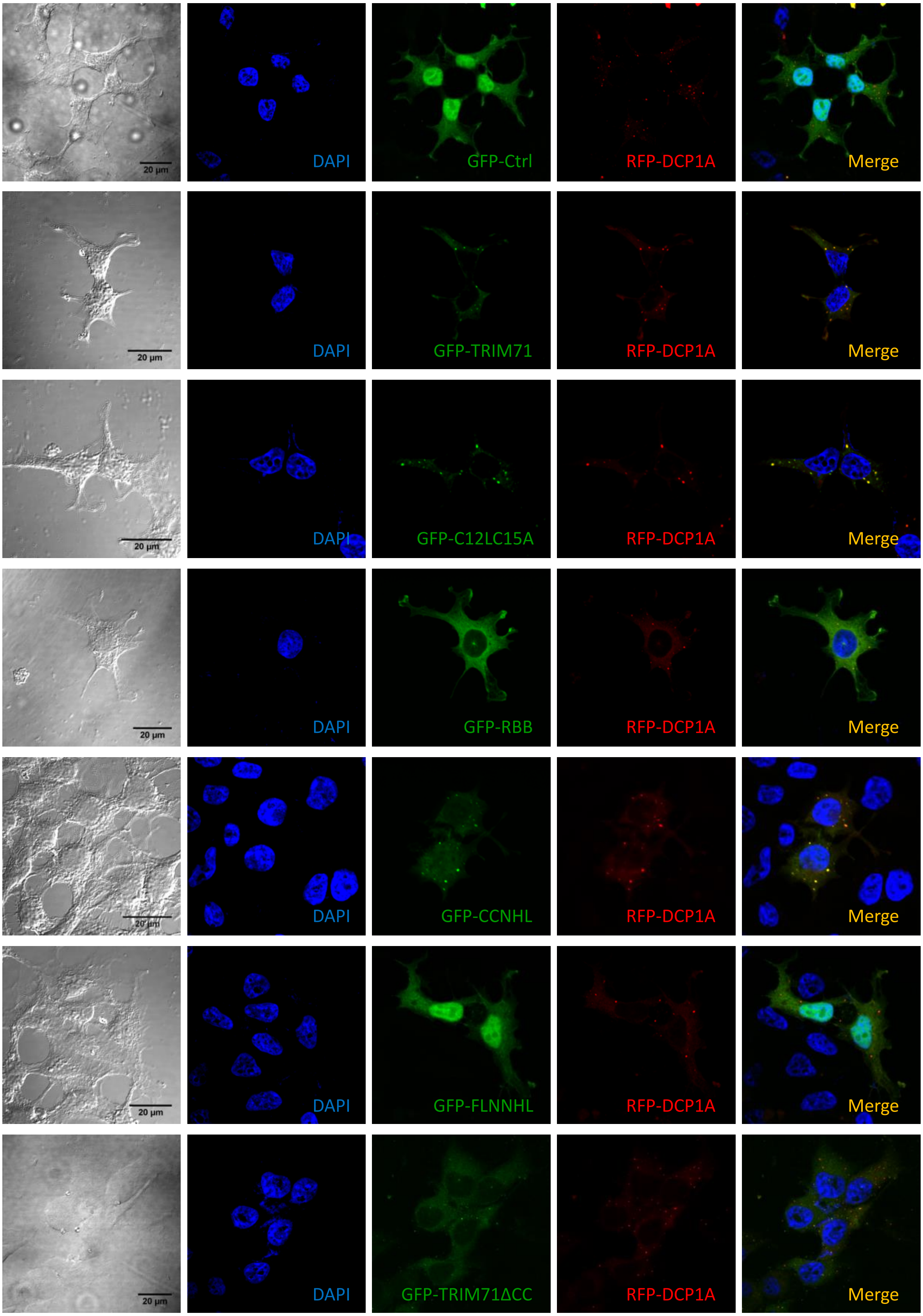

Supplementary Figure 6 (Related to Fig. 4): Representative confocal laser scanning microscopy images showing the localization of the specified GFP-TRIM71 constructs (green) relative to the specific P-body marker RFP-DCP1A (red) in transiently transfected HEK293T cells. A total of 50 cells per condition were observed for GFP-RFP colocalization to calculate the percentage of cells in which each construct localized within P-bodies, depicted in main Fig. 4E.

Figure S7 (Related to Fig. 5)

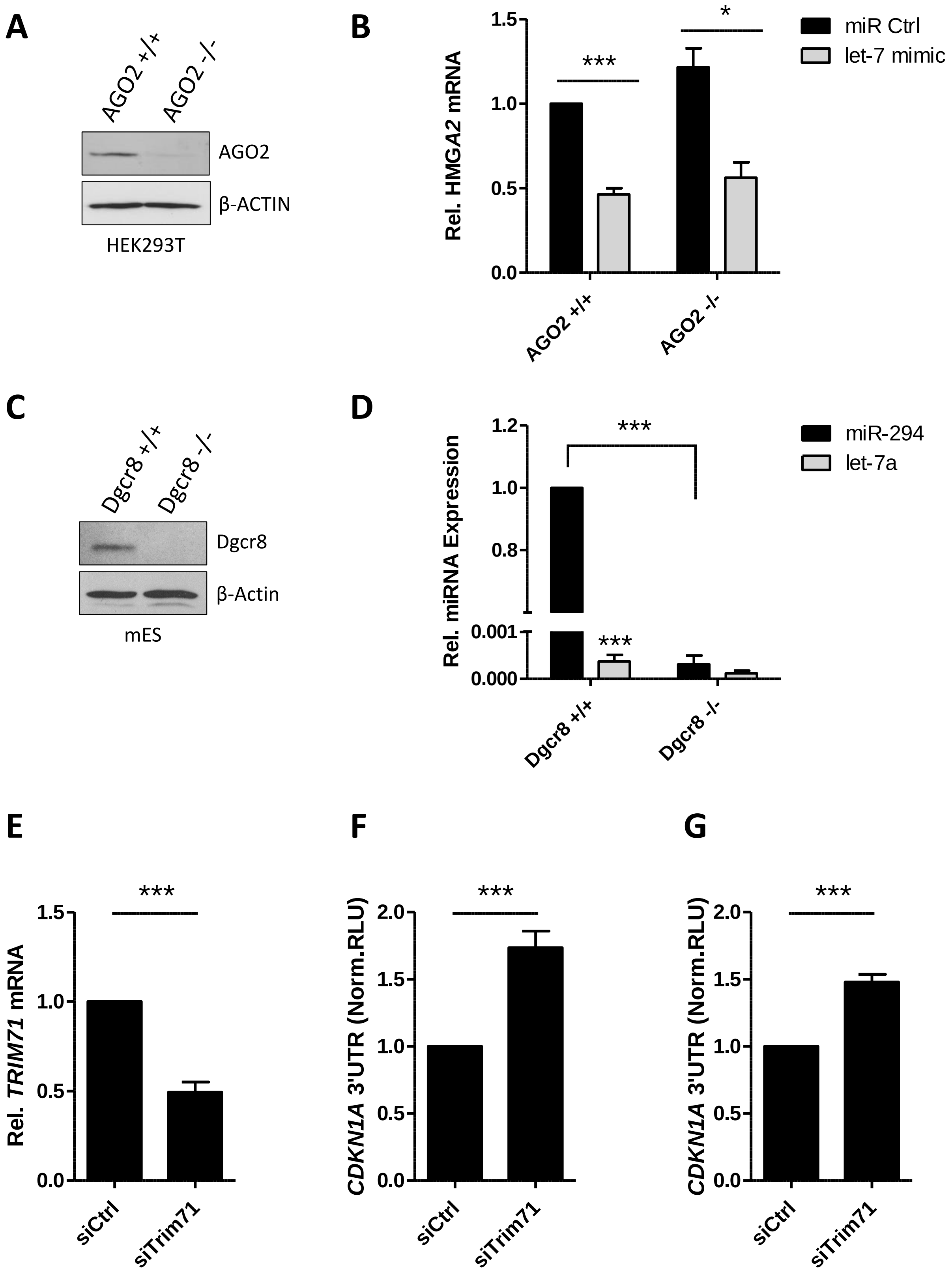

Supplementary Figure 7 (Related to Fig. 5): A) Immunoblot showing AGO2 protein levels in wild type (AGO2 +/+) or AGO2 knockout (AGO2 -/-) HEK293T cells. **B)** qPCR measurements of the let-7 *bona fide* mRNA target HMGA2 upon miRCtrl or Let-7 miRNA mimic overexpression in wild type (AGO2 +/+) or AGO2 knockout (AGO2 -/-) HEK293T cells (n=3). **C)** Immunoblot showing Dgcr8 protein levels in wild type (Dgcr8+/+) or Dgcr8 knockout (Dgcr8-/-) mES cells. **D)** qPCR measurements of the stem cell specific miR-294 and the differentiation promoting miR let-7a in wild type (Dgcr8+/+) or Dgcr8 knockout (Dgcr8-/-) mES cells (n=3). **E)** qPCR measurements of TRIM71 mRNA upon TRIM71 knockdown (siTrim71) in mES cells (n=6). **F)** Derepression of the full length CDKN1A 3'UTR luciferase reporter upon TRIM71 knockdown in wild type (Dgcr8+/+) or **G)** Dgcr8 knockout (Dgcr8-/-) mES cells (n=3-4). For qPCRs of mRNA and miRNAs, HPRT1 housekeeping gene and U6 snRNA were used for normalization, respectively. Norm. RLU = Normalized Relative Light Units. Graphs represent Mean±SEM. Statistical significance was calculated with a two-tailed unpaired t test: *pvalue<0.05; ***pvalue<0.005.

Figure S8 (Related to Fig. 6)

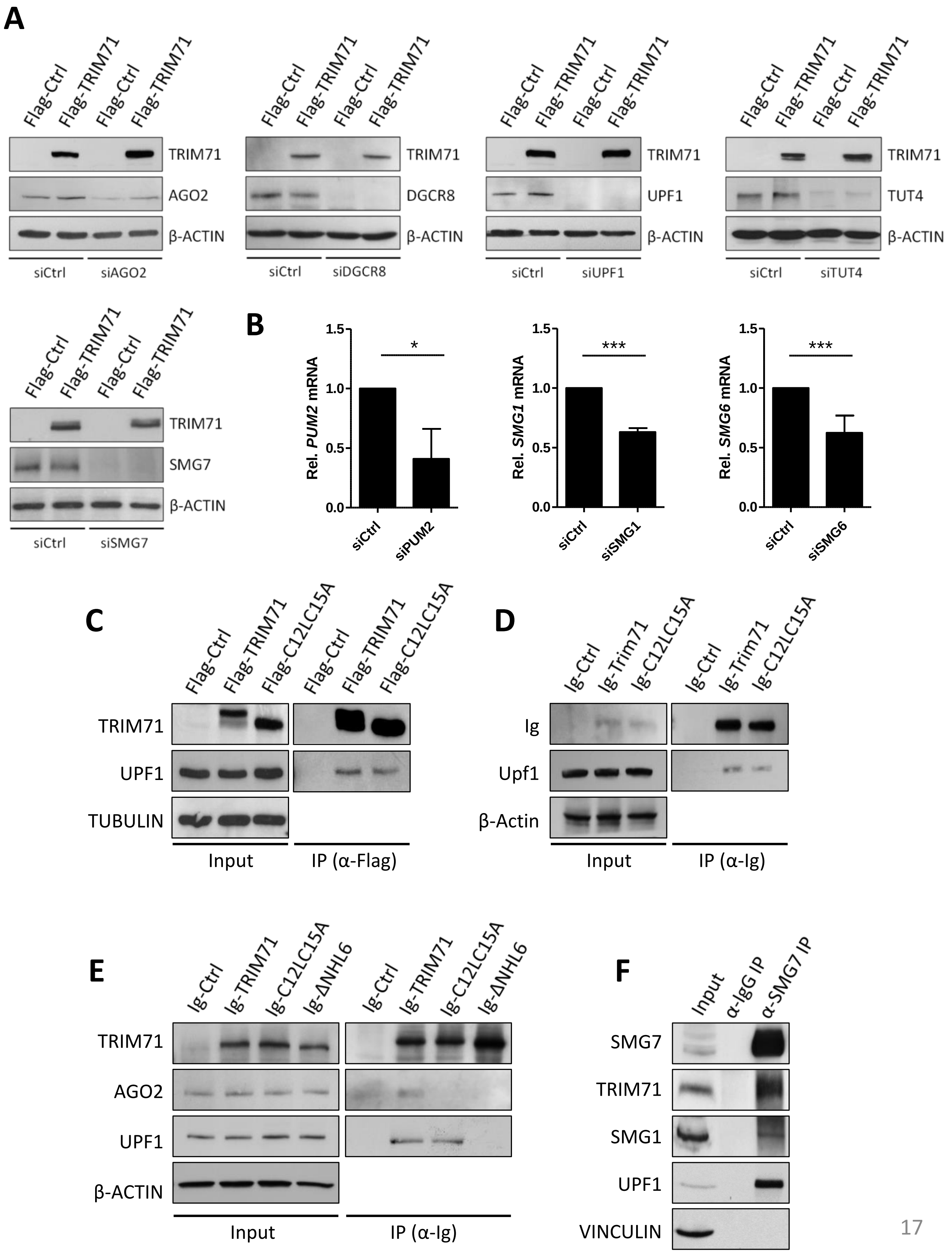

Supplementary Figure 8 (Relative to Fig. 6): A) Immunoblots or **B)** mRNA quantification by qPCR showing knockdown levels of specific P-body proteins, corresponding to luciferase experiments of Fig. 6A, B, F and G. Graphs represent Mean±SD (n=3-4). Statistical significance was calculated with a two-tailed unpaired t test: *pvalue<0.05; ***pvalue<0.005. **C)** Representative immunoblot showing endogenous UPF1 coprecipitation with Flag-TRIM71 wild type and ubiquitylation mutant (Flag-C12LC15A) in HEK293T cells. **D)** Representative immunoblot showing endogenous UPF1 coprecipitation with Ig-Trim71 and Ig-C12LC15A in mES cells. **E)** Representative immunoblot showing UPF1 and AGO2 coprecipitation capability with Ig-Ctrl/Ig-TRIM71/Ig-C12LC15A/Ig-ΔNHL6 in HEK293T cells. **F)** Immunoblot showing the coprecipitation of endogenous TRIM71, UPF1 and SMG1 with endogenous SMG7 in HepG2 cells.

Figure S9 (Relative to Fig.7)

A

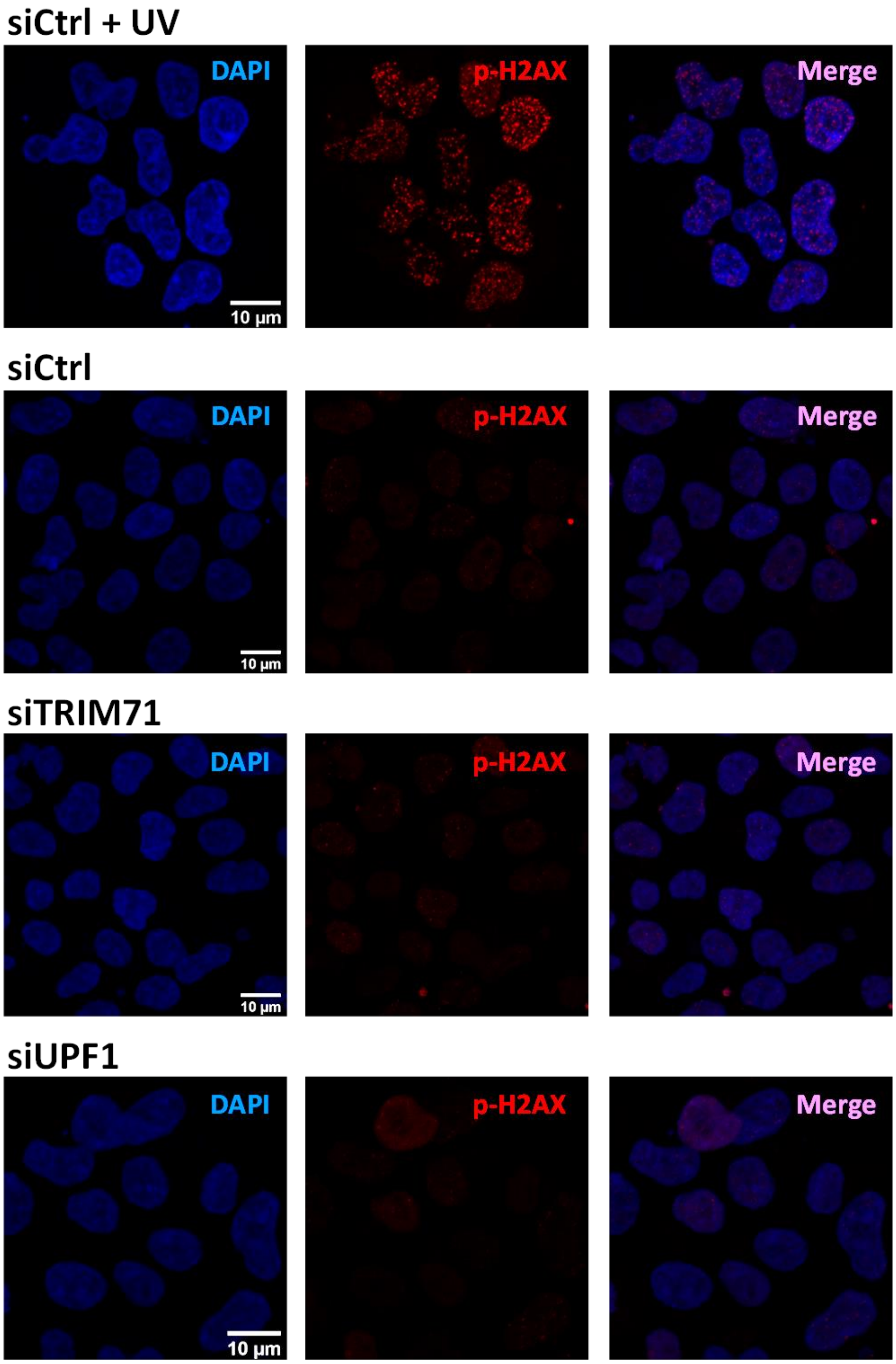

A

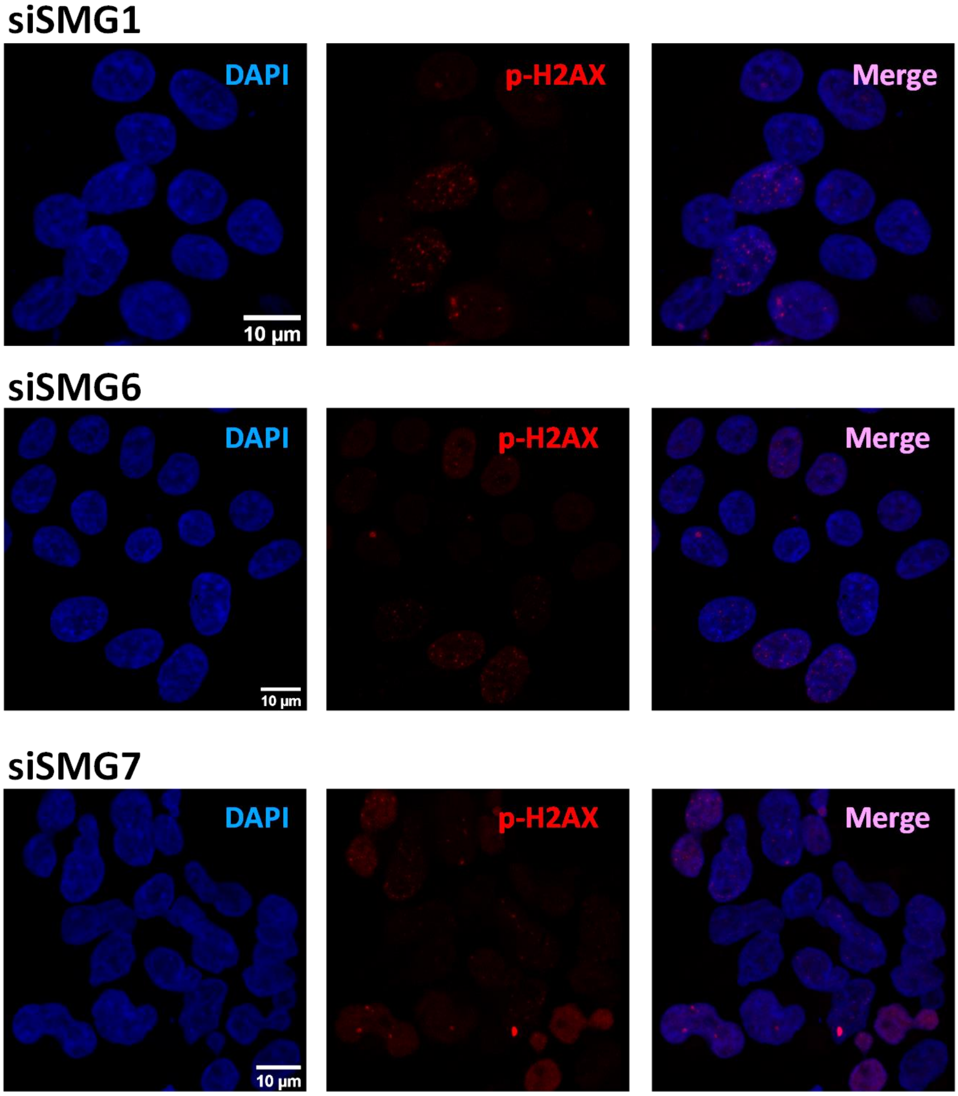
siSMG1
DAPI
p-H2AX
Merge
10 µm
siSMG6
DAPI
p-H2AX
Merge
10 µm
siSMG7
DAPI
p-H2AX
Merge
10 µm

B

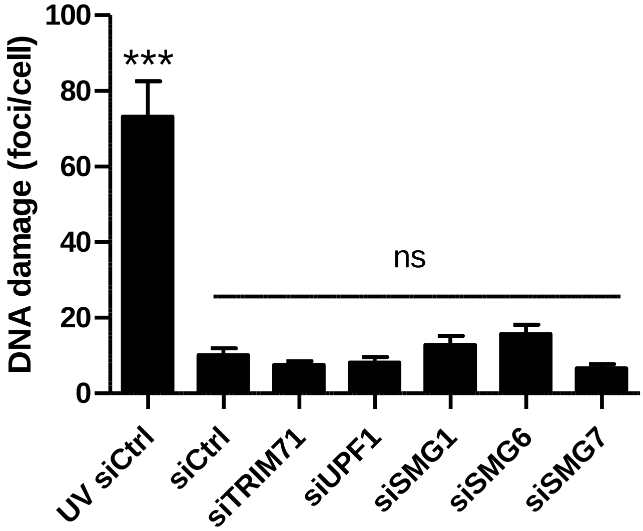
DNA damage (foci/cell)
100
80
60
40
20
0
***
ns
UV siCtrl
siCtrl
siTRIM71
siUPF1
siSMG1
siSMG6
siSMG7

C

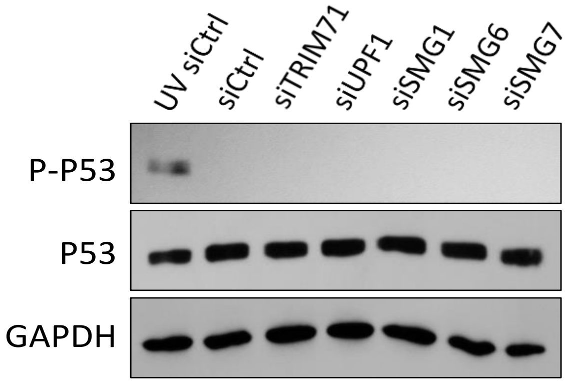

D

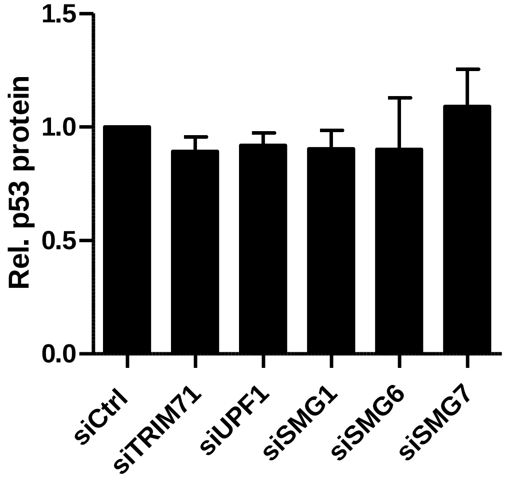

E

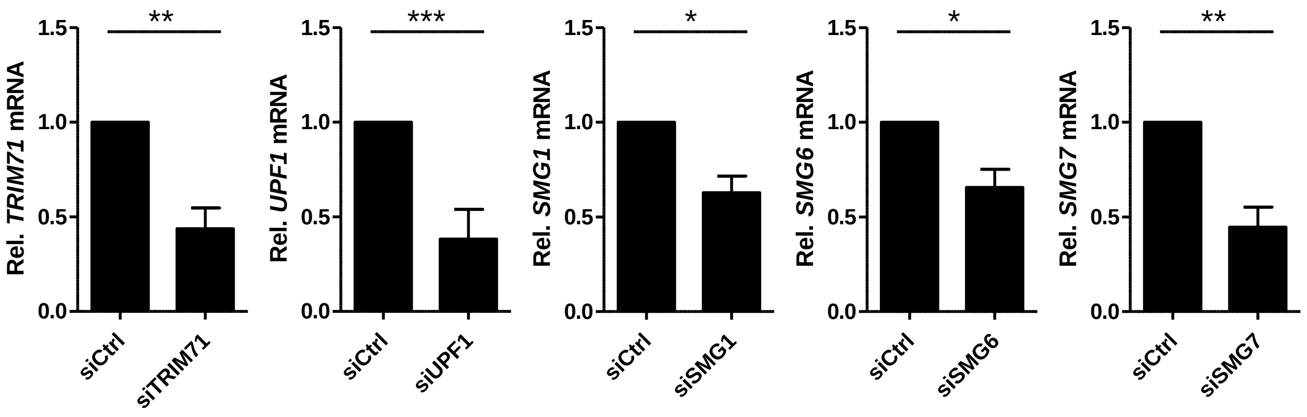

Supplementary Figure 9 (Relative to Fig. 7): A) Confocal microscopy maximal intensity projection images (stacked) showing phospho-H2AX (Ser139) staining in HEK293T cells upon TRIM71 and NMD components knockdowns. A control sample was UV-irradiated with 100 J/cm^2 to induce DNA damage i.e. high H2AX phosohorylation levels. **B)** Quantification of phospho-H2AX staining (foci per cell) as a measure for DNA damage in at least 50 cells per condition. Gaph represents Mean±SEM. Statistical significance was calculated with a two-tailed unpaired t test between each sample and the non-irradiated siCtrl sample: ns = non-significant; ***pvalue<0.005. **C)** Representative immunoblot showing levels of p53 and phospho-p53 in samples used for A and B. **D)** Quantification of p53 protein band densitometry in a blot replicates of C (n=4). **E)** qPCR quantification of the indicated mRNAs to control the knockdown levels of TRIM71 and NMD components for all experiments depicted in A-D. Graphs represent Mean±SD (n=3-4). Statistical significance was calculated with a two-tailed unpaired t test: *pvalue<0.05; **pvalue<0.01; ***pvalue<0.005.

Figure S10 (Related to Fig. 7)

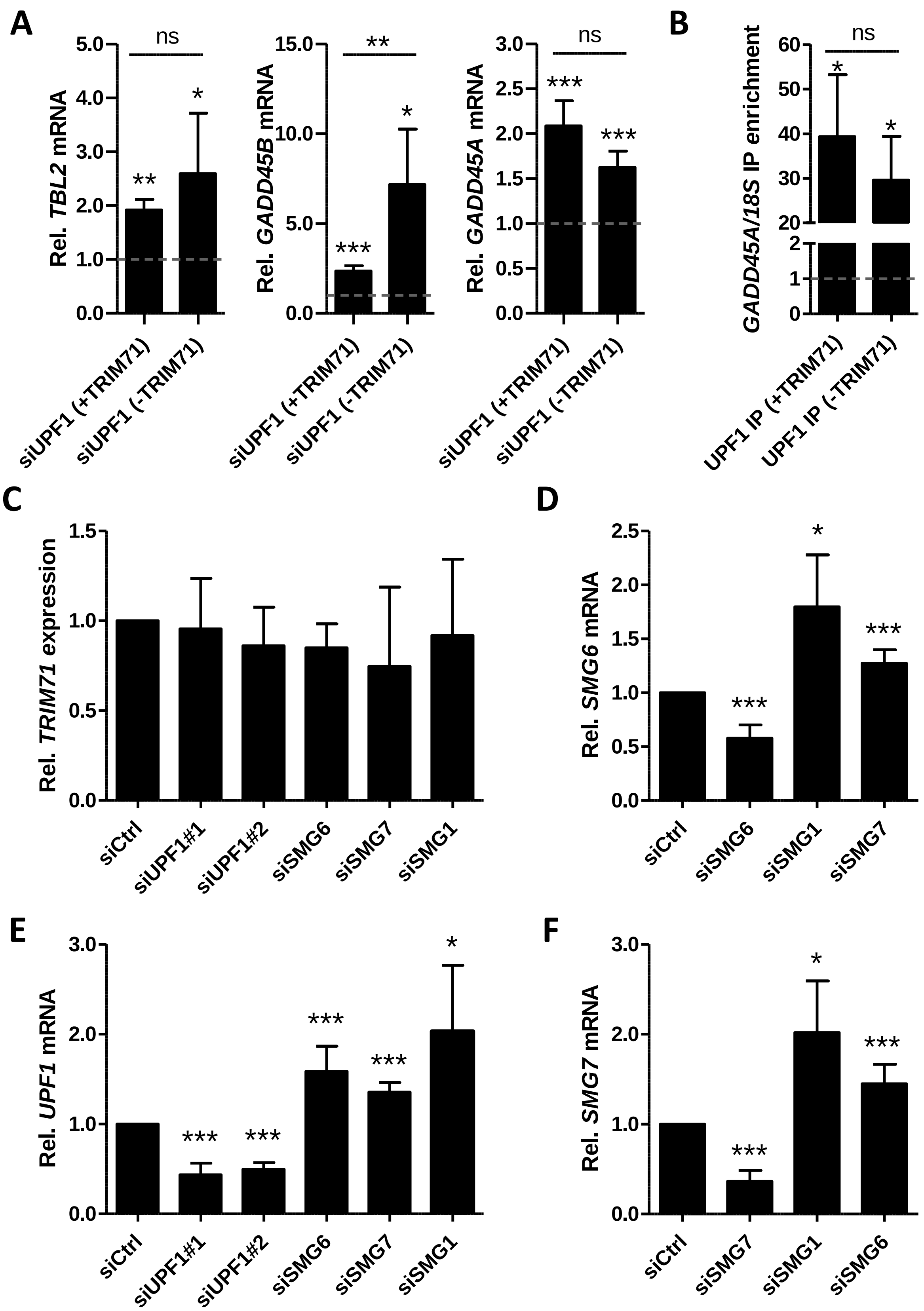

Supplementary Figure 10 (Relative to Fig. 7): A) qPCR quantification of NMD canonical targets upon UPF1 knockdown (siUPF1) in the presence(+) and absence(-) of TRIM71, achieved by TRIM71 knockdown in HEK293T cells (depicted in main Fig. 7C) which express naturally low amounts of TRIM71 (n=3-8). **B)** qPCR quantification of GADD45A/18S IP enrichment of upon RNA-IP with Flag-UPF1 (UPF1 IP) overexpressed in HEK293T cells in the presence (+) and absence (-) of TRIM71 (n=3-4). **C)** qPCR quantification of TRIM71 **D)** SMG6 **E)** UPF1 and **F)** SMG7 mRNA expression levels upon knockdown of the indicated NMD proteins, demonstrating that the cross-regulation observed between different NMD components does not apply to TRIM71 (n=4-5). Graphs represent Mean±SD. Statistical significance was calculated with a two-tailed unpaired t test: ns = non-significant. *pvalue<0.05; **pvalue<0.01; ***pvalue<0.005.

Figure S11 (Relative to Fig. 8)

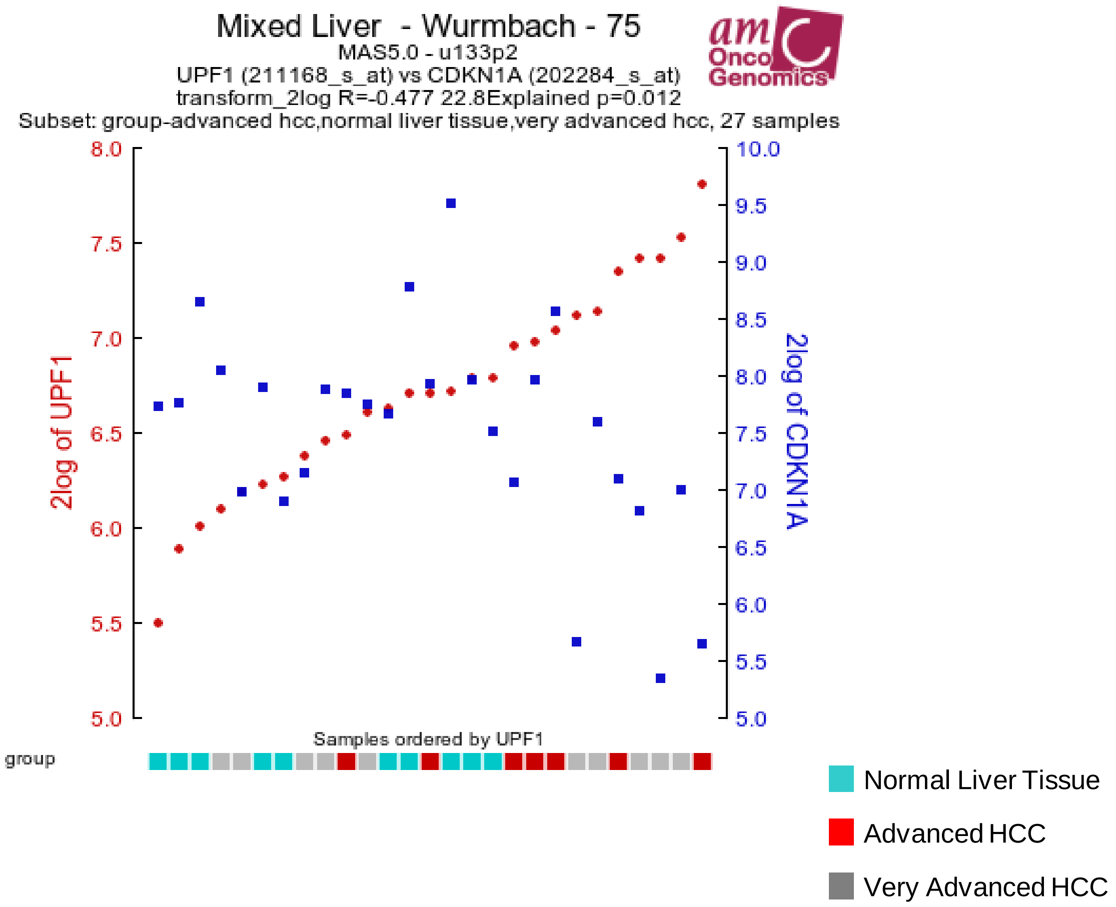

Supplementary Figure 11 (Related to Fig. 8): Negative correlation between UPF1 and CDKN1A mRNA expression in samples from advanced stage-HCC patients (Source: R2 Genomics Analysis and Visualization Platform (http://r2.amc.nl), Wurmbach Dataset).

Figure S12 (Relative to Fig. 8)

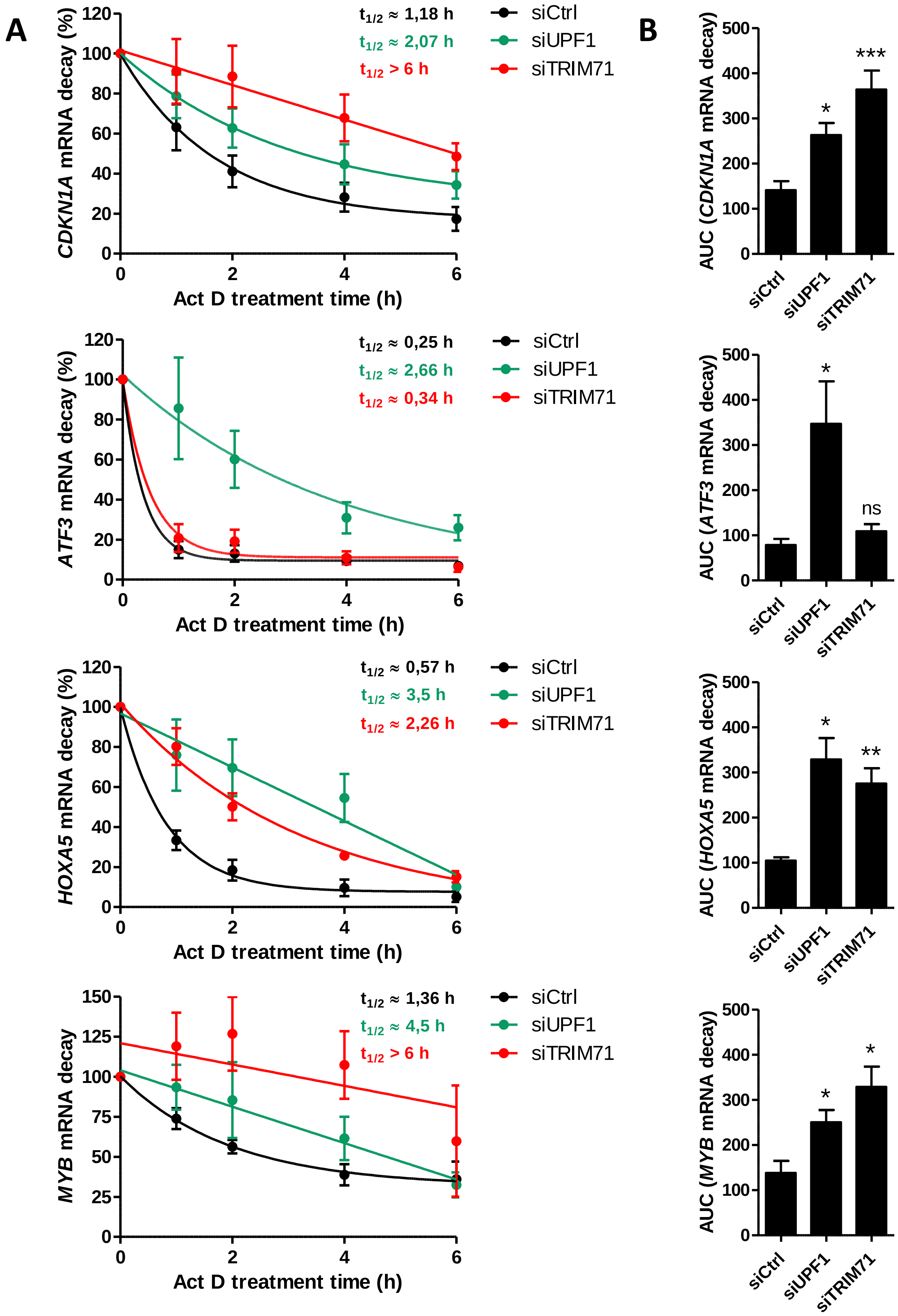

Supplementary Figure 12 (Related to Fig. 8): A) mRNA decay of the indicated mRNA targets in control, UPF1 knockdown and TRIM71 knockdown HepG2 cells, 1h, 2h, 4h and 6h after transcriptional inhibition with Actinomycin D. mRNA decay is depicted as percentage (%) relative to the initial amount of mRNA quantified in the DMSO control (time point 0h). Decay curves were adjusted by non-linear regression with GraphPad Prism7 to calculate the mRNA half-life for each target ($t_{1/2}$) as a measurement of mRNA stability. Graphs represent Mean±SEM (n=3-4). **B)** Area under curve measurements (AUC, units = % mRNA*h) of the individual decay curves whose average curves are depicted in A, calculated with GraphPad Prism7. Statistical significance was calculated with a two-tailed unpaired t test: *pvalue<0.05; **pvalue<0.01; ***pvalue<0.005.

Figure S13

A

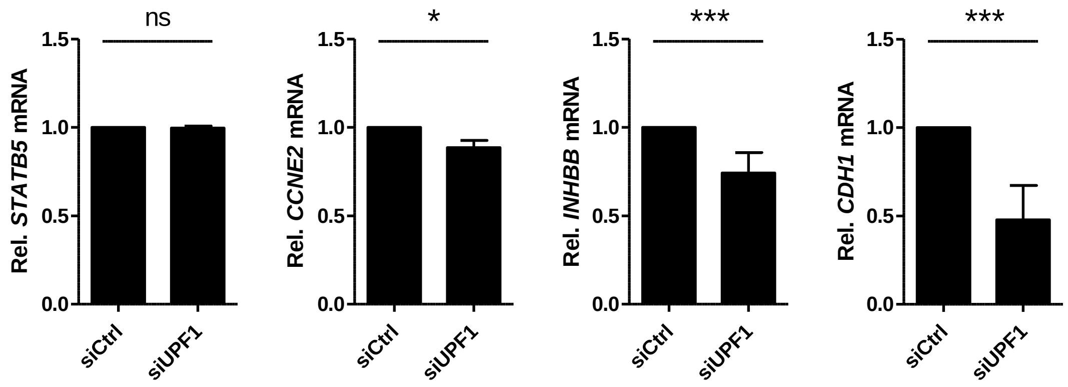

B

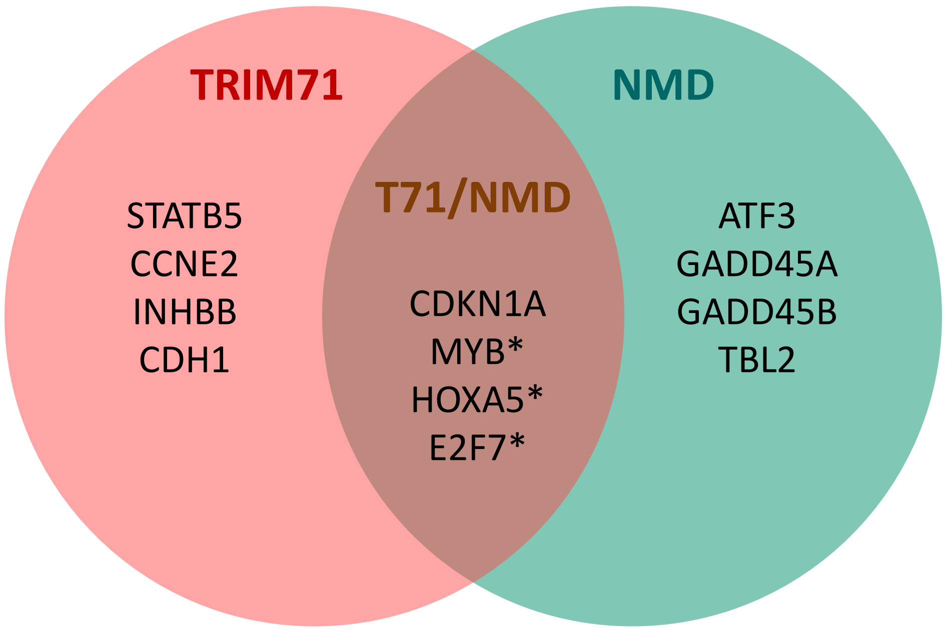

Supplementary Figure 13: A) qPCR quantification of known TRIM71 targets upon UPF1 knockdown in HEK293T cells, showing that some TRIM71 targets are not upregulated by NMD impairment via UPF1 knockdown. Graphs represent Mean ±SD (n=3-4). Statistical significance was calculated with a two-tailed unpaired t test: *pvalue<0.05; **pvalue<0.01; ***pvalue<0.005. **B)** Venn Diagram showing the regulation of mRNA targets used in this study by either NMD-only, TRIM71-only (from results depicted in A) or by the TRIM71/NMD axis identified by our work. *These targets were regulated by a TRIM71/UPF1-dependent mechanism.
